# Inconsistent and incomplete retraction of published research: A cross-sectional study on Covid-19 retractions and recommendations to mitigate risks for research, policy and practice

**DOI:** 10.1371/journal.pone.0258935

**Published:** 2021-10-27

**Authors:** Geoff Frampton, Lois Woods, David Alexander Scott

**Affiliations:** Southampton Health Technology Assessments Centre (SHTAC), Wessex Institute, Faculty of Medicine, University of Southampton, Southampton, United Kingdom; Stellenbosch University and Kenya Medical Research Institute, KENYA

## Abstract

**Background:**

Retraction of published research can reduce the dissemination of incorrect or misleading information, but concerns have been raised about the clarity and rigor of the retraction process. Failure to clearly and consistently retract research has several risks, for example discredited or erroneous research may inform health research studies (e.g. clinical trials), policies and practices, potentially rendering these unreliable.

**Objective:**

To investigate consistency and clarity of research retraction, based on a case study of retracted Covid-19 research.

**Study design:**

A cross-sectional study of retracted Covid-19 articles reporting empirical research findings, based on searches of Medline, Embase and Scopus on 10^th^ July and 19^th^ December 2020.

**Key results:**

We included 46 retracted Covid-19 articles. The number eligible for inclusion nearly doubled, from 26 to 46, in five months. Most articles (67%) were retracted from scientific journals and the remainder from preprint servers. Key findings: (1) reasons for retraction were not reported in 33% (15/46) of cases; (2) time from publication to retraction could not be determined in 43% (20/46) of cases; (3) More than half (59%) of retracted Covid-19 articles (27/46) remained available as original unmarked electronic documents after retraction (33% as full text and 26% as an abstract only). Sources of articles post-retraction were preprint servers, ResearchGate and, less commonly, websites including PubMed Central and the World Health Organization. A retracted journal article which controversially claimed a link between 5G technology and Covid-19 remains available in its original full text from at least 60 different websites.

**Conclusions:**

The retraction process is inconsistent and often ambiguous, with more than half of retracted Covid-19 research articles remaining available, unmarked, from a wide range of online sources. There is an urgent need to improve guidance on the retraction process and to extend this to cover preprint servers. We provide structured recommendations to address these concerns and to reduce the risks that arise when retracted research is inappropriately cited.

## Introduction

Retraction of published research is essential for correcting errors in the scientific literature and maintaining trust in research [1–4]. Published research may be retracted for various reasons, including misconduct (fraud, plagiarism, duplicate publication), honest error (mistakes, legitimate artefacts or re-interpretation of data in light of new facts), author disputes, ethical issues, or failure to comply with publishing standards [5–15].

The proportion of published research articles that have been retracted increased steadily during the 1970s to 2000s, across a wide range of scholarly disciplines [1, 2, 5–8, 10, 16], but the rate of increase slowed after 2012 [1, 2]. The frequency of retraction reflects a balance between the rate at which flawed or erroneous research is published and the extent to which the scientific community (authors and their institutions, editors and publishers, and readers of the research) can detect and act upon inappropriate publications. Retraction rates have been found to vary with the scholarly discipline [8, 16], journal impact factor [3, 4, 6, 14] and country of authorship [5, 7].

Retraction was first listed as a specific publication category in Medline in 1980, and the National Library of Medicine has provided bibliographic indexing of retracted articles with links to their retraction notices since 1984. Web of Science has listed retracted articles since around 2000; and the International Committee of Medical Journal Editors (ICMJE) have recommended since at least 1997 that a retraction notice should be provided clearly stating the reason for retraction [10]. However, studies investigating retractions during the 1980s to 2010s found that the reasons for retraction were ambiguous or not reported for 5% to 20% of retracted articles [4, 5, 7, 8, 10–12, 14, 15, 17–19]. Furthermore, many retracted articles remained available post-retraction without being marked as retracted [4, 10, 17, 18, 20–22] and retracted articles have been cited in the academic literature post-retraction without acknowledgement of their retracted status [3, 7, 8, 12, 14, 23–27]. The time from publication to retraction has varied considerably, ranging from several months to more than 10 years [5, 7, 8, 12–15, 18], and occasionally more than 20 years [5]. Studies have also shown that retraction policies and the clarity and consistency of retraction notices have varied widely across academic journals [4, 6, 7, 10, 19, 28]. Retraction of research may be requested by authors, editors, journals, publishers, or other parties such as an author’s institution. The majority of retractions (59% to 81%) assessed were made by authors [10, 12, 15], whilst the person or organisation responsible for retraction was unclear or not reported in 5% to 53% of retractions [10, 15, 19].

In response to these inconsistencies the Committee on Publication Ethics (COPE) published guidelines in 2009 to improve consistency and clarity of the retraction process [29]. The COPE guidelines state that notices of retraction should clearly identify the retracted article and be linked with it; the retraction notices should be available freely, state the reason for retraction and who is retracting; and publications should be retracted as soon as possible after the editor is concerned that a publication is seriously flawed. The COPE guidelines also state that “prompt retraction should minimise the number of researchers who cite the erroneous work, act on its findings, or draw incorrect conclusions, such as from ‘double counting’ redundant publications in meta-analyses or similar instances” [29]. In a further development to improve understanding of the retraction process and promote good practice, a not-for-profit organisation, Retraction Watch, was launched in 2010 and now hosts a database of over 80,000 retracted scientific articles, as well as blogs discussing key articles and issues with retractions [30].

The Covid-19 pandemic has resulted in an enormous proliferation of scientific research and scientific articles [31, 32]. For instance, searches for Covid-19 articles on PubMed found that the publication rate had already reached 59 articles per day by late April 2020 [33], with almost 10 published every working hour by early May 2020 [32]. To date, more than 100,000 COVID-19 articles have been produced [34]. Concerns have been raised that the “rush to publish” Covid-19 research has led to a decline in scientific rigor, resulting in the mass publication of poor quality research [32, 35], creating extensive research waste [36], and leading to inappropriate and harmful information dissemination and decision making [37]. A number of high-profile Covid-19 studies have been retracted after publication [37–44].

Retraction of research is associated with several risks, including that discredited or erroneous work may continue to be cited, and may inform health research studies (e.g. clinical trials), policies and practices, as well as causing research waste if research, policies or practices have to be suspended or corrected. Examples of consequences arising from retractions of Covid-19 research are provided in S1 Appendix.

When reading the scientific literature on Covid-19 we came across several anomalies in the retraction of Covid-19 research studies whereby we were unable to reconcile information on why and/or when some retractions had taken place. Several studies investigating the characteristics of retracted Covid-19 research have also reported ambiguity and inconsistency of the retraction process [38, 40, 43–45]. However, the prevalence of these issues is unclear. We therefore conducted a cross-sectional study of the Covid-19 articles that had been retracted up to 19^th^ December 2020, to clarify the extent of inconsistencies in the process for retracting Covid-19 research and whether any recommendations could be made to address them. We focused on Covid-19 retractions because (i) the relatively high rate of retractions provides a contemporaneous and sizeable cohort for analysis, and (ii) retractions could have important implications for decision-making in a pandemic if not communicated clearly and consistently.

## Materials and methods

### Research question, aim and objectives

Our research question is “How consistent and transparent is the process for retracting Covid-19 research from scientific journals and preprint servers?”. Our aim was to investigate the consistency and transparency of the process for retracting Covid-19 research articles, based on a representative sample of publication sources, to help determine whether any specific changes in the retraction process are necessary. We focused on issues relating to the retraction process that occur once a decision has been made to retract an article, i.e. we did not investigate ways to prevent retractions (which would require a different type of research study). Our specific objectives were (1) to investigate the completeness of reporting of the process for retracting COVID-19 articles and the accessibility of articles after retraction during 2020; and (2) if necessary and appropriate, to provide recommendations on how specific aspects of the retraction process could be improved.

### Methodological approach

We conducted a cross-sectional study of Covid-19 research articles retracted during 2020. To ensure objectivity and reduce the risk of introducing bias in our methods, we conducted a systematic search to identify a representative sample of the retracted articles; specified inclusion criteria that the articles should meet; required at least two researchers to assess each included article; and employed an iterative approach to data extraction (details below).

### Searches

We conducted searches for retracted articles in Ovid Medline, Ovid Embase and Scopus bibliographic databases on 10^th^ July and 19^th^ December 2020. We used a combination of key terms, and MeSH and EMTREE index terms, relating to retraction, combined with Covid-19 terms from published Covid-19 search strings (the EPPI Centre’s Covid-19 living systematic map of the evidence [46] search strategy for Medline; and a modified version of the Centers for Disease Control and Prevention Library search string for Covid-19 for Scopus [47]. Our Medline search strategy is shown in S2 Appendix.

### Article selection

Many of the Covid-19 articles have been posted on preprint servers [31, 39, 48–52]. Given the increasing importance of preprint servers as a means of promptly disseminating research we included retractions both from academic journals and preprint servers.

Articles were eligible for inclusion in our analysis if they met all of the following criteria: (1) The article had Covid-19 as its main focus. (2) The article had been made available either on a preprint server or was published in a scientific journal but had been subsequently retracted (described either as retracted or withdrawn). Articles which were described as “accepted” by journals and were available online as full text were eligible. (3) The article reported empirical research, was a review of empirical research, or reported case studies or case series. Letters, commentaries and opinion papers were excluded unless they reported a description of the methods and results of empirical research. We also excluded a retracted fake paper on Covid-19 that had been written specifically to test the rigor of a suspected predatory journal’s peer review and editorial practices [53].

We included articles classified as a “temporary removal”, provided that the article had not been reinstated or replaced by the time of our final assessment (19^th^ December 2020).

### Data extraction

Each of the eligible studies for analysis was read by the research team (GF, LW, DAS) and a draft table of study characteristics was prepared as a data extraction template. The template was modified iteratively as relevant information from the studies was encountered on each read. The final template (S3 and S4 Appendices) was then approved by the research team and applied to all the included studies to ensure consistency of the extracted data. One researcher (GF or LW) extracted data into the template and this was then checked by a different researcher (LW or DAS). Any disagreements in interpretation were resolved through consultation between all researchers.

### Data analysis

We conducted a descriptive analysis to explore characteristics and accessibility of the retracted Covid-19 articles, by tabulating and summarising subsets of the extracted data recorded in the data extraction template (S3 and S4 Appendices).

## Results

We identified 26 relevant retracted Covid-19 articles from our search on 10^th^ July 2020 and a further 20 unique articles from our search on 19^th^ December 2020, i.e. the number of eligible articles had nearly doubled in a 5-month period, giving a total of 46 retracted articles in total for analysis. Our searches gave very similar results to those of Retraction Watch [54]: we included only two articles [55, 56] that were not in the Retraction Watch list at 19^th^ December 2020.

### Characteristics of the retracted articles

Thirty-one articles (67%) were published in 31 unique scientific journals and 15 were published on the preprint servers bioRxiv, [57] medRxiv [52] and SSRN [48] (S3 Appendix). Of the 31 articles published by scientific journals, 8 were retracted by the authors, 14 were retracted by the journal (i.e. the editors and/or publisher), 6 were retracted by an unspecified combination of the authors and journal, while the retractor was unclear for 3 articles. Where reported, the articles hosted on preprint servers were retracted by the authors (Table 1).

**Table 1:** Reasons for the retraction of COVID-19 research papers up to 19^th^ December 2020.

Reason for retraction	Publication location and retractor						Total
	Published on preprint server, retracted by authors	Published on preprint server, retractor unclear	Published in journal, retracted by authors	Published in journal, retracted by journal ^a^	Published in journal, retracted by “authors and/or journal” ^a^	Published in journal, retractor unclear	
***No information given***	2 [70, 71]	1 [72]	1 [73]	3 [74–76]	5 [77–81]	3 [82–84]	**15**
***Issues relating to misconduct or noncompliance with standards***							**10**
Data veracity concerns not resolved	-	-	2 [65, 66]	-	-	-	2
Objectives not approved by the local IRB	2 [85, 86]	-	-	-	-	-	2
Lack of full author consent to publish	1 [87]	-	-	-	-	-	1
Lack of consent from data owner	1 [88]	-	-	-	-	-	1
Manipulation of peer review (no details given)	-	-	-	1 [89]	-	-	1
Violation of publication ethics	-	-	-	1 [90]	-	-	1
Privacy issues regarding study conduct (contested by author)	1 [91]	-	-	-	-	-	1
Reuse of copyrighted questionnaire without permission	-	-	-	1 [92]	-	-	1
***To update article with new information***							**9**
To conduct further verification experiments	2 [93, 94]	-	-	-	-	-	2
To revise paper based on peer review	2 [95, 96]	-	-	-	-	-	2
To revise paper with more recent data	2 [97, 98]	-	-	-	-	-	2
Further research needed	-	-	-	1 [68]	-	-	1
Authors decided their opinions expressed were incomplete	-	-	1 [99]	-	-	-	1
Due to “controversy about HCQ and potential changes to results after peer review”	1 [100]	-	-	-	-	-	1
***Errors in data or analysis***							**4**
Inaccurate reporting of outcome LOD	-	-	-	1 [101]	-	-	1
Major flaw in methodology (authors should have used T cells instead of T-cell lines)	-	-	1 [102]	-	-	-	1
Suspected false positive result	-	-	1 [103]	-	-	-	1
Major error in data entry for analysis	-	-	1 [55]	-	-	-	1
***Duplicate publication***							**7**
Duplicate publication	-	-	-	5 [59, 60, 62–64]	1 [58]	-	6
Plagiarism	-	-	-	1 [61]	-	-	1
***Other issues***							**1**
Failure to demonstrate first mortality case ^b^	-	-	1 [69]	-	-	-	1
**Total**	**14**	**1**	**8**	**14**	**6**	**3**	**46**

HCQ: Hydroxychloroquine; IRB: Institutional review board; LOD: Limit of detection.

^a^ refers to the editor(s), editorial board and/or publisher.

^b^ the authors retracted their case study when they realised that they were not the first to report the first case of COVID-19 death during pregnancy.

### Characteristics of retractions

Characteristics of the retractions are summarised in Table 1. Seven articles were retracted due to duplication or plagiarism [58–64]. The remaining 39 articles (85%) each reported a unique study.

Papers were retracted for a wide range of reasons. Where reported, the reasons for retraction included some relatively minor issues as well as major problems with study conduct or data analysis. Thus, the “weight” of a retraction (i.e. it’s importance in relation to ensuring good standards of research rigor) differed across studies.

#### Unclear reasons for retraction (*N* = 15)

COPE guidelines state that the reason for retraction should always be reported [29]. However, for 15 of the 46 Covid-19 retractions (33%) this information was not provided (Table 1). Scientific journals rather than preprint servers were responsible for most of these unclear retractions (12 and 3 retractions respectively).

#### Retraction due to misconduct and noncompliance with standards (*N* = 10)

Ten retractions (22%) were related to research misconduct or noncompliance with research or publication standards (other than plagiarism or duplication). These included two high-profile studies by Mehra et al. [65, 66] retracted from the Lancet and New England Journal of Medicine (NEJM) due to concerns about the credibility of the Surgisphere company which had supplied the datasets for both studies [41, 67]. The articles retracted due to issues of misconduct or noncompliance with standards were equally split (5 each) between preprint servers and scientific journals (4 articles).

#### Retraction to update an article with new information (*N* = 9)

Nine retractions (20%) were stated to have been retracted to enable an article to be updated with new information, for example based on comments received during peer review. At the time of our final assessment (19^th^ December 2020) none of these articles had been replaced and it is unclear whether, and when, they would be updated. Most (7/9) of these articles were retracted from preprint servers and in nearly all (8/9) cases it was the article authors who had retracted them. The one retraction requested by a scientific journal was retracted due to concerns that the conclusions were based on theory not field epidemiology data and thus further research was needed [68].

#### Retraction due to errors in data or analyses (*N* = 4)

Four articles (9%) were retracted due to errors in data or analyses, all of which had initially been published in scientific journals, although in 3 cases it was the article authors who requested the retraction. It is unclear how the authors came to be aware of these errors.

#### Retraction due to duplicate publication or plagiarism (*N* = 7)

Six articles were duplicates of previously published papers, [58–60, 62–64] and one [61] was retracted due to plagiarism, all of which were retracted by journals. Two of the duplicate articles reflect self-plagiarism [58, 59] whilst the remaining four were accidental repeat duplications by four different journals [60, 62–64].

#### Other reasons for retraction (*N* = 1)

One article on COVID-19 was retracted from a journal on the authors’ request because the authors became aware that their paper was not the first to document mortality in a pregnant COVID-19 patient [69]. Instead of deleting one line from their abstract to correct this (“To our knowledge, this is the first report of maternal death with confirmed COVID-19 infection”) they retracted the full article.

### Time from publication to retraction

The time period between publication of an article and its retraction was calculable for only 26 of the 46 retracted articles (57%) (S4 Appendix). Where available, the median time from publication to retraction was longer for articles published in scientific journals (45.5 days; range 7 to 217 days; n = 14) than for those published on preprint servers (10.5 days; range 9 to 34 days; n = 12). Three of the four articles published on the bioRxiv preprint server were retracted after only one day, [87] two days [96] and four days [88]. This very short timescale from publication to retraction suggests lack of adequate preparation of manuscripts, perhaps reflecting the lack of detailed author guidelines for preprint servers.

### Availability of original research articles after retraction

Of the 46 retracted Covid-19 articles, 27 (59%) continued to be available as original unmarked versions from online sources after they had been retracted (Table 2). Fifteen articles (33%) remained available as original full-text articles whilst the remaining 12 (26%) were available only as abstracts. The host preprint servers where the articles had originally been published (medRxiv, bioRxiv and SSRN) and the ResearchGate academic networking platform were the key sources of these original full text articles and abstracts (for further details see S3 Appendix). Some articles which had been retracted relatively early in the course of the Covid-19 pandemic, in January and February 2020 [87, 96, 97], had been available as original unmarked full-text versions for more than 8 months after retraction at our check on 19^th^ December 2020 (Table 2).

**Table 2:** Availability of retracted COVID-19 articles as unmarked original full-text papers and/or abstracts after retraction.

Retracted article	Retraction notice date	Original (unmarked) full text available at 19^th^ Dec 2020	Original (unmarked) abstract available at 19^th^ Dec 2020 (shown only for articles where full text not available)
Adjodah et al. [98]	23 Oct 2020	No	Yes: WHO COVID literature collection
Alhowary et al. [80]	Unclear	Yes: ResearchGate	-
An et al. [55]	Jul/Aug 2020	No	Yes: Host journal & ResearchGate
Bility et al. [79]	8^th^ Oct 2020	Yes: ResearchGate	-
Cercy [91]	21^st^ Jun 2020	Yes: Host preprint server & ResearchGate	-
Chen et al. [87]	28^th^ Jan 2020	Yes: Host preprint server & ResearchGate	-
Chu et al. [93]	15^th^ Apr 2020	No	Yes: ResearchGate
Davido et al. [95]	20^th^ May 2020	Yes: Host preprint server	-
Dumantepe et al. [73]	24^th^ Aug 2020	No	Yes: Other source
Etemadifar et al. [77]	15^th^ Sep 2020	No	Yes: Other source
Fioranelli et al. [89]	Jul 2020	Yes: at least 60 online sources (S5 Appendix)	-
Gaibazzi et al. [85]	20^th^ Jun 2020	Yes: Host preprint server & ResearchGate	-
Gaibazzi et al. [86]	13^th^ Jun 2020	Yes: Host preprint server	-
Irshad et al. [92]	unclear	Yes: PubMed Central	-
Kanwar [75]	unclear	No	Yes: Other source
Karami et al. [69]	May 2020 ^a^	No	Yes: ResearchGate
Kim et al. [100]	14^th^ Jun 2020	Yes: Host preprint server	-
Mulvey et al. [60]	Jun 2020	No	Yes: ResearchGate
Patel et al. [71]	Unclear ^a^	Yes: Other source	-
Pradhan et al. [96]	2^nd^ Feb 2020	Yes: ResearchGate	-
Raharusuna et al. [72]	Unclear ^a^	Yes: Other source	-
Siyu et al. [94]	7^th^ Mar 2020	Yes: Host preprint server & ResearchGate	-
Vavougios [78]	15 Jul 2020	No	Yes: ResearchGate
Wang et al. [83]	Unclear ^a^	No	Yes: ResearchGate (Chinese)
Wei et al. [81]	Unclear	No	Yes: ResearchGate
Yang et al. [97]	21^st^ Feb 2020	Yes: Host preprint server	-
Zhang et al. [76]	unclear	No	Yes: ResearchGate

^a^ information sourced from Retraction Watch

The availability of full text articles and abstracts shown in Table 2 is a snapshot of a dynamic situation. For example, a search of Google Scholar showed that a journal article on “5G Technology and induction of coronavirus in skin cells” by Fioranelli et al. 2020 [89] retracted on 24^th^ July 2020 was still available on 5^th^ September 2020 from up to 47 different online sources, which increased to 60 online sources by 19^th^ December 2020. This article remained available from 60 online sources at 26^th^ June 2021, although three of the original sources were no longer available whilst a further three new online sources emerged (S5 Appendix). The Fioranelli article is highly controversial, claiming an unsubstantiated notion that 5G Technology influences Covid-19 infection.

In some cases unmarked original versions of retracted articles remained available post-retraction at reputable online sites: a full-text article that had been removed from the journal website continued to be available from PubMed Central (PMC) [56] (though not available from the sister PubMed site), whilst two retracted abstracts continued to be available in the WHO COVID Literature Collection [104, 105].

For eight of the 12 retracted articles that had been published on the bioRxiv and medRxiv preprint servers [85–87, 91, 94, 95, 97, 100], clicking on the “Download PDF” link gave access to an unmarked original version of the full text. Moreover, for three articles the “Info/History” tab on these preprint servers provided several different unmarked original versions of the full text article [85–87].

### Inappropriate citation of retracted articles

Due to the relatively short timescale of our study we could not investigate the citation patterns of retracted Covid-19 research. However, we noted that an editorial published in the NEJM [106] continued to cite a study [66] that had been retracted from the same journal on 4^th^ June 2020, with the editorial remaining uncorrected at 26^th^ June 2021, more than 12 months after the retraction.

## Discussion and recommendations

We identified several problems with the way that Covid-19 articles have been retracted, notably lack of clarity on the timing of and reasons for retractions, and continued availability of retracted articles, often from multiple sources. Concerns had already been raised across a range of scholarly disciplines prior to the Covid-19 pandemic regarding ambiguity in the reasons for retraction [4, 5, 7, 8, 10–12, 14, 15, 17–19], continued availability of published research after retraction [4, 10, 17, 18, 20–22] and continued citation of retracted research [3, 7, 8, 12, 14, 23–27], suggesting that COPE guidelines were not adequately adhered to prior to 2020. However, these studies did not compare retractions before and after the COPE guidelines [29] were published and so the impact of the guidelines is difficult to determine. Our findings, together with those of other studies on Covid-19 retractions [38–40, 44], indicate that improvements in adherence to retraction guidelines are urgently needed.

The underlying reasons for the continued lack of clarity and consistency in the retraction of Covid-19 journal articles are unclear but could in part reflect inadequate awareness of the existence of the COPE guidelines, and inadequate editorial oversight of the large amounts of Covid-19 research published during the pandemic [39, 107]. The lack of clarity and consistency in the retraction of articles from preprint servers is perhaps not surprising since there are no guidelines on the process for retracting articles from preprint servers (the COPE guidelines [29] refer only to retractions from academic journals). Since preprint servers have become an important route for disseminating Covid-19 research ideas and findings [48–51] there is a need for guidance on retractions to be extended to cover preprint servers.

### Risks of incomplete and ambiguous Covid-19 retractions

Continued availability of research articles after retraction is a serious problem because it creates uncertainty as to which version(s) of published articles can be trusted, potentially undermining scientific research and public confidence in medicine and other sciences. During the Covid-19 pandemic, stakeholders including the public, news organisations and scientists have had to make sense of rapidly-emerging and often contradictory information in order to make critical decisions whilst inaccurate information has also been circulating, e.g. on social media channels and the Internet. The paper by Fioranelli et al. illustrates the ease with which inaccurate information can be disseminated by failing to completely retract discredited ideas (S5 Appendix).

Retraction can have serious consequences, depending on the nature of the retracted information and the impact that it has on end-users of the research. Retracted articles may inform health agency guidance, systematic reviews, meta-analyses, or prediction models, potentially rendering their conclusions and recommendations unreliable. Clinical research (e.g. controlled trials) may be suspended or adjusted based on findings from retracted articles, with a range of implications for patients, investigators and health agencies. Retracted articles may be described in detail in newspapers or other popular media, potentially disseminating unreliable information to the public. And retractions can in turn trigger further retractions or corrections (“domino effect”), potentially creating confusion around which versions of publications are reliable. Examples of each of these issues and consequences arising from the retraction of Covid-19 articles are provided in S1 Appendix.

### Key retraction problems and solutions

#### Incomplete retraction: The problem

The continued availability of more than half of the retracted COVID-19 articles as unmarked pre-retraction versions defeats the purpose of the retraction process, which is to prevent the dissemination of discredited or unreliable science [29]. As illustrated by the links in S5 Appendix, unmarked original versions of retracted articles shared by online sites may be downloaded directly via a search engine, bypassing any contextual information at the host websites. The COPE guidelines do not provide any specific recommendations to prevent the dissemination of articles after they have been retracted.

#### Incomplete retraction: Solutions

All copies of a retracted article, including both the abstract and the full text (and any accompanying materials), should be clearly marked as retracted [50]. A prudent marking approach for retracted articles would be to use a strong (e.g. bold red) watermark stating “retracted” or “withdrawn” that diagonally covers each full page of the article so that the retraction status of all parts of an article cannot be in doubt (several of the retracted articles included in our study provide examples of this [58, 59, 61, 65, 101]). Clear watermarking of all retracted articles is a logical failsafe to ensure that the retracted status of an article will remain clear if it becomes detached from its retraction notice or contextual information at a host website.

Once a decision has been made to retract an article, it should be the authors’ responsibility to ensure that all copies of the article documents (abstract, full text, supplementary material) that they have made available are clearly marked as retracted. This applies to copies of the article on preprint servers and websites, including personal websites, ResearchGate and other academic networking platforms, as well as any copies of the original article stored on a computer. Journal editors should check that any linked documents, such as editorials, are updated as necessary to reflect the retraction. Ideally, editors (or at a broader level publishers) should have a standard operating procedure to ensure that no documents are missed from consideration when an article is retracted.

#### Unclear reasons for retraction: The problem

One third of the retracted Covid-19 articles did not report the reason for retraction, which prevents lessons from being learnt, and could lead to the unnecessary repetition of errors. Failure to disclose the reasons for retraction makes it more difficult to identify and ameliorate misconduct.

Lack of explanation of the reasons for retraction also creates ambiguity around which versions of an article can be trusted, particularly if an article is retracted temporarily and later reinstated. For example, an article reporting on factors associated with SARS-CoV-2 infection [84] was temporarily removed from the Journal of Hospital Infection and later reinstated (S3 Appendix) with the same citation details but no explanation of why the article had been retracted, when the reinstatement was made, or whether the reinstated version differed in any way from the retracted version. This could lead to two different versions of the article in circulation which would be indistinguishable in bibliographies.

#### Unclear reasons for retraction: Solutions

The COPE guidelines [29] recommend that the “reason(s) for retraction” should be stated, and that the retraction notice should “be objective, factual, and avoid inflammatory language” [29]. We understand that some publishers currently appear to have a contrary policy to this. For example, Elsevier does not require authors to provide a reason when requesting retraction of articles from their scientific journals, and we note that Elsevier had the largest market share of the journal articles from which COVID-19 articles had been retracted (S4 Appendix). Publishers should ensure that the reason for the retraction is published in the retraction notice.

The COPE guidelines state that retraction notices should be linked to retracted articles where possible but do not state the reverse, i.e. that retracted articles should also be linked to their retraction notices. Publishers of articles should, ideally, ensure that a retracted article is directly linked to its retraction notice, e.g. via an embedded hyperlink.

#### Unclear timing of retractions: The problem

We were unable to determine the retraction date for 43% (20/46) of the retracted Covid-19 articles (S4 Appendix). For these articles it is not possible to accurately determine either the time from publication to retraction or the time that an article remains available post-retraction.

Perhaps unsurprisingly given their relatively large market share of the journals included in our study, the publisher Elsevier accounted for around half of the retracted articles for which the date of retraction was not reported (S4 Appendix), although five articles retracted from journals published by Elsevier did report the retraction date, suggesting an inconsistent policy.

#### Unclear timing of retractions: Solutions

The COPE guidelines [29] state that “notices of retraction should be published promptly to minimise harmful effects” but they do not specify that the date of the retraction and of the retraction notice should be provided. The date of publication and date of retraction should both be clearly stated, alongside the reason for retraction, in the retraction notice posted by a scientific journal or on a preprint server.

### General recommendations on retraction from the pre-Covid-19 literature

As well as the COPE guidelines published in 2009, prior to the Covid-19 pandemic several recommendations had been made regarding how the retraction process should be improved, and our findings suggest that these recommendations remain pertinent:

- Better mechanisms are needed for raising researchers’ awareness of retracted literature [8, 14], including better systems for linking retractions to their retraction notices [6].
- Scientists and their students should develop the habit of looking for corrections or retractions of works they cite in their publications [25]. Journals should require prospective authors to declare that they have checked their manuscript’s reference list for retracted articles [108]. Computer software should be implemented to check reference lists for retracted articles free of charge in any database or the internet [24].
- A better vocabulary for talking about retractions is needed and a database for classifying retractions would be useful [6]. Standardisation of the wording of retraction notices may be helpful [3], for example using a standard retraction form with a checklist of retraction reasons [11].
- Automated methods to deliver article status updates (such as CrossMark) may reduce persistence of error in the scientific literature [22].
- Retracted articles should be cited appropriately, with negative (i.e. critical) citation encouraged [9].
- General reforms that have been suggested include increasing the responsibility of journal editors; and providing greater transparency and clarity around mistakes in research [6]. An example of how journals could take ownership of their retraction data and use self-critical reflection to improve standards is provided by Fang et al. [3].

### Recommendations on retraction based on our findings

Below we provide comprehensive recommendations on good practice for the retraction of published articles, bringing together the findings from our study on Covid-19 retractions, recommendations made by other authors prior to the Covid-19 pandemic summarised above, and the COPE guidelines on retraction [29]. These recommendations are broadly applicable, not limited to Covid-19 research, and cover articles published on preprint servers as well as those published in academic journals.

For any recommendations to be acted upon, the relevant stakeholders (authors, preprint managers, journal editors and journal publishers, as well as the wider scientific community, including authors of reviews, meta-analyses and guidelines) must first be aware of the problem. It is important therefore that the limitations in the retraction process are widely communicated. Our recommendations are more detailed, focused and extensive than those provided by the COPE guidelines and we hope that they will be useful to the Committee on Publication Ethics and other stakeholders when updating future retraction guidelines.

#### Recommendations for study authors, journal editors and publishers, and preprint managers

Table 3 provides our recommendations for (i) authors of retracted articles, (ii) journal editors and publishers, and (iii) preprint server managers to address the specific problems of (a) continued availability of retracted articles, specifically the need to ensure that all retracted articles are clearly marked as such; (b) lack of reporting of the reasons for retraction; (c) lack of reporting of the date of retraction; and (d) ambiguity in who retracted an article.

**Table 3:** Recommendations for study authors, journal editors and publishers, and preprint managers.

Problem	Recommendations for authors of retracted articles	Recommendations for journal editors and publishers	Recommendations for preprint server managers
**A high proportion of retracted COVID-10 articles remain available unmarked after retraction, undermining the purpose of retraction**	1) Identify all online locations (preprint servers, websites, ResearchGate, journals, etc) where copies of the article had been placed	1) Once a retraction has been agreed, ensure that all documents relating to the retracted article, including any copies of the abstract, full text and supplementary material that will be published by the journal are prominently marked as retracted.	1) Check that all retracted documents are prominently marked as retracted, including the abstract, supplementary material, and all previous versions where these remain available on the preprint server (e.g. under the “Info/History” and “Full Text” tabs of bioRxiv and medRxiv preprint servers). Preferably use full page strong watermark on each page to avoid ambiguity whether any parts of an article are retracted.
	2) Check whether all retracted documents (abstracts, full text, supplementary material) at these locations are clearly marked as retracted.		
		2) Check whether there are any editorials or commentaries in the journal that cite the retracted article. If necessary, update these to ensure the retraction status of the original article is unambiguous and editorial or commentary conclusions remain valid.	
	3) Prominently mark any retracted documents that had not been marked as retracted. If necessary, contact journal editor, preprint server manager or website manager to facilitate document access and/or permission to make these amendments		
**Reasons for retraction are not reported in a proportion of articles (we estimate one third of retracted COVID-19 articles); the identity of retractors is ambiguous in a proportion of articles (we estimate 29% of retracted Covid-19 articles)**	1) When retracting an article from a preprint server ensure that the reason for retraction is provided and the identity of the stakeholders involved in the retraction is explained so that this can be stated in the retraction notice.	1) Editors should ascertain the reason for retraction from the study authors if necessary and report this in the retraction notice together with the roles of all stakeholders involved in the retraction. Where this is not standard journal policy, consultation with the publisher may be helpful to ensure consistency.	1) When authors request retraction of an article, ensure that the reason for retraction is provided by the authors and clearly stated in the retraction notice. Preprint servers such as SSRN which currently do not provide a retraction notice should include one.
	2) When retracting an article from a scientific journal, if it is not journal policy to publish the reason for retraction, request that the editor makes an exception for consistency with COPE guidelines [29].		
**The date of retraction is not reported in a proportion of articles (we estimate around 40% of retracted COVID-19 studies)**	1) Ensure that the date of retraction is provided on all retracted documents and agrees with the date on which the retracted documents were marked as retracted.		

#### Recommendations for all authors of articles, including authors of reviews, meta-analyses, guidelines and prediction models

Table 4 provides our recommendations for authors of all articles.

**Table 4:** Recommendations for authors of all articles, including (but not limited to) authors of reviews, meta-analyses, guidelines and prediction models.

Problem	Recommendation	Examples of possible approaches
**Risk of including retracted articles in scientific papers (including but not limited to reviews, meta-analyses, guidelines or prediction models)**	When preparing a new document for submission to a scientific journal or preprint server ensure that none of the articles cited in the document have been retracted (NB This should apply to the numerous Covid-19 systematic reviews that have been registered as protocols or are underway [109]). Readers of papers should check whether any cited articles were retracted subsequent to the paper’s publication.	If an active CrossMark button [110] is available on the electronic version of the article, use this to check retraction status. Alternatively, consult the Retraction Watch database or COVID-19 retractions list [54]; or conduct a search for “retracted” or “withdrawn” articles, e.g. by running a search in Google Scholar, PubMed or several databases if necessary (for an example search strategy see S2 Appendix). If available use bibliographic management software (e.g. Zotero [111]) that can detect retracted articles.

The COPE guidelines on retraction [29] acknowledge that secondary research articles, including systematic reviews and meta-analyses may themselves need to be corrected or retracted if they include retracted studies, but as far as we are aware there is no agreed process for ensuring that this happens in a consistent way, or indeed at all. Scientific journals should require authors to demonstrate that they have checked for any retractions when preparing their manuscript.

To ensure transparency in systematic reviews and meta-analyses, any articles that are otherwise eligible but excluded due to having been retracted should be specified as retracted in the list of excluded studies when following standard PRISMA [112] principles.

### Future research needs

#### Raise awareness of research retraction issues

An extensive literature has developed in relation to limitations of the retraction process, but to our knowledge the critical problem that most retracted COVID-19 articles continue to be accessible as unmarked original versions of the abstract or full text is not widely known. It is important therefore to raise awareness of this problem, to support the recommendations to reduce dissemination of retracted research.

#### Encourage the checking of retraction status

The feasibility of developing common standards for encouraging authors to check for retractions before submitting their manuscript for publication should be investigated. Existing reporting checklists, such as CONSORT [113], STROBE [114], SPIRIT [115], PRISMA [112] and others [116] could be updated to require authors to declare that they have checked for retractions when preparing their manuscript. We note that Retraction Watch is currently listed as a resource by Cochrane Training [117] but there is not a requirement that authors of reviews should check that none of their included studies and cited articles have been retracted.

#### Guidelines for retractions from preprint servers and other online sources

Given the proliferation of preprint servers and their popularity as sources of scientific publication [31, 39, 48–51] a set of good practice publishing standards should be established for preprint servers and the Internet. We understand that the COPE has a broad remit that covers the spectrum of scientific publication, not limited to academic journals, and it seems logical for the COPE guidelines on retraction to be extended to these online publication sources. However, as our study confirms, some of the existing COPE guidelines are not consistently followed and it is imperative that the reasons for this are investigated and understood in order to improve the likelihood of future compliance. Furthermore, as discussed above, current COPE guidelines are insufficiently detailed in some areas.

Preprints are seen as an important way of soliciting open peer review and scientific journals have been working increasingly in collaboration with preprint servers to improve the peer review of scientific research prior to publication [52, 118–120]. Of the 100 top-ranked clinical journals, 86% now allow authors to submit their articles to preprint servers prior to publication in the journal [50]. Journals which work in association with preprint servers could have a role to play in helping to ensure that appropriate guidelines for retraction are applied by the preprint servers that they are linked with.

#### Link articles to information on their retraction status

As illustrated by the hyperlinks in S5 Appendix, unmarked electronic versions of retracted articles can easily be downloaded without needing to visit the host website for any contextual information. Many researchers may never see retractions or corrections because they just download the electronic copies of the articles they need and never again consult the original source [6]. This problem was recognised over a decade ago and was one of the factors that led to the development of the CrossMark button for electronic articles [6]. Clicking on an article’s CrossMark button gives the reader instant access to information on whether the content has been updated, corrected or retracted [110] and saves readers time compared to the other ways of checking retractions shown in Table 4. Unfortunately, CrossMark is not available on many electronic articles. Initiatives to promote wider uptake of CrossMark, or other information linking systems for electronic articles are needed.

### Limitations of this study

Our recommendations concern good practice in handling retractions once a decision to retract an article has been made; but they do not address ways to avoid the need for retractions in the first place, such as by ensuring that reporting standards are adhered to prior to publication [121] or by optimising peer review [43], as this is beyond the scope of our study. A list of legitimate reasons for retracting articles is provided in the COPE guidelines on retraction [29].

The increasing frequency of COVID-19 retractions means that our study, which was up to date at 19^th^ December 2020, could be quickly superseded with new retractions [122]. However, the critical finding, that most of the retracted COVID-19 articles continue to be available after retraction, would remain valid unless a substantial rapid change in the behaviour of authors, preprint server managers, journal editors and publishers occurs. This seems very unlikely in the absence of general awareness of the problem and a current lack of recommendations to address it [54].

Continued citation of retracted articles was a well-known problem prior to the Covid-19 pandemic [3, 5, 7–9, 14, 18, 20, 23, 24, 26, 27], with some articles being cited for more than a decade after their retraction [20, 24, 27]. However, we were unable to include a meaningful analysis of citation patterns in our study due to the relatively short time that has elapsed since Covid-19 articles were retracted and the unclear timing of retractions for 43% of the articles.

Finally, our study was not based on a pre-specified and peer-reviewed protocol. This was due to the limited timescale we had available to conduct the work on this fast-moving topic. We protected our methods from becoming overly subjective or biased in the absence of a protocol by using an iterative approach to data extraction to ensure all articles were assessed consistently and fairly. All extracted data were checked by at least two researchers to minimise subjectivity and errors, and all data supporting our conclusions and recommendations are provided in the accompanying Tables and Appendices together with references and links to their original sources.

## Conclusions

More than half of retracted Covid-19 articles remain available, unmarked, after retraction, from a wide range of online sources. The process for retracting covid-19 research is inconsistent and often unclear, with key information about the reasons for and timing of retraction frequently missing or ambiguous. Our findings suggest an urgent need to improve guidance on the retraction process, such as updating the COPE guidelines, and extending guidance to cover retractions from preprint servers as well as from journals. We provide targeted evidence-based recommendations to help authors of retracted articles, journal editors and publishers, and preprint server managers to achieve this. We also provide recommendations for authors of any research documents on how to reduce the risk of inappropriately citing retracted articles. Our case study was on Covid-19 retractions but our findings are consistent with those of studies in other scholarly disciplines and our recommendations are therefore applicable generally, not limited to Covid-19 research.

## Supporting information

S1 Appendix Examples of research waste and other consequences of Covid-19 retractions. (PDF)

S2 Appendix Medline search strategy. (PDF)

S3 Appendix Characteristics of retracted Covid-19 articles. (PDF)

S4 Appendix Timing of publication and retraction of Covid-19 articles. (PDF)

S5 Appendix Sources of unmarked copies of the retracted paper by Fioranelli et al [89]. (PDF)
