# Supplementary material for: Inconsistent and incomplete retraction of published research: A cross-sectional study on Covid-19 retractions and recommendations to mitigate risks for research, policy and practice
Source: PLoS One. 2021 Oct 27;16(10):e0258935. doi: 10.1371/journal.pone.0258935 (PMC8550405; doi:10.1371/journal.pone.0258935)
Supplement: S1 Appendix — (PDF) [file pone.0258935.s001.pdf]

S1 APPENDIX Examples of research waste and other consequences of Covid-19 retractions

Depending on the nature of the retracted information and the impact that it has on end-users of the research, retraction of COVID-19 research can have serious consequences. Examples of key risks arising from the retraction of Covid-19 articles are as follows:

Retracted studies inform health agency guidance on COVID-19

A retracted study linked to the Surgisphere organisation inappropriately claimed that the antiparasitic drug ivermectin dramatically reduced the need for mechanical ventilation and reduced COVID-19 mortality [70], which led to a surge in demand for the drug across Latin America, with health agencies in several countries allowing ivermectin to be given widely to COVID-19 patients [41, 67]. Another study linked to Surgisphere reported effects of cardiovascular disease, ACE inhibitors and angiotensin receptor blockers on COVID-19 mortality and was cited by World Health Organization (WHO) guidance on smoking and COVID-19 (later updated to remove the citation) [123]. A study reporting an analysis of infection rates among close contacts of COVID-19 patients [68] had influenced decisions made by the US White House Coronavirus Task Force before being retracted [124].

Retracted studies inform systematic reviews, meta-analyses, or prediction models

One of the studies linked to Surgisphere [66] was included in (at least) two meta-analyses of angiotensin-converting enzyme inhibitors (ACEIs) and angiotensin receptor blockers (ACEs) [125, 126] (one of these meta-analyses [125] was subsequently updated by independent researchers [127] to exclude the retracted article). A retracted article on epidemiological and clinical features of COVID-19 in China [97] was cited in a COVID-19 prediction model published in Lancet Infectious Diseases [128] and in an influential COVID-19 epidemiological modelling report published by Imperial College [129] (both were later corrected).

Clinical research (e.g. controlled trials) is suspended or adjusted

The WHO temporarily suspended the hydroxychloroquine arm of the SOLIDARITY trial for COVID-19 [130] and several other COVID-19 hydroxychloroquine trials in different countries were also put on hold [67] as a result of the findings from one of the Surgisphere studies [65] which was later retracted from The Lancet journal. Suspension of clinical research substantially inconveniences patients, clinicians, and policy makers as well as wasting patients' and clinicians' time and money, and creating failed targets, evidence gaps, possible harm, and other ethical issues.

Retracted studies are described in detail in newspapers or other popular media

A retracted article relating to social distancing on public transport [74] was reported in graphic detail by the South China Morning Post [131] and in the UK Daily Mail [132]. The South China Morning Post article was shared more than 53,000 times on social media; after the study was retracted the newspaper reported this but the report received less social media interest [49]. A further retracted paper about glycemic control in Type 1 diabetes during lockdown [90] was cited in detail by Medscape, a popular website aimed at health professionals (a note on the retraction was later added by Medscape) [133]. Such dissemination of retracted research has potential to confuse the public, clinicians, and policy makers, and may lead to inappropriate decision making.

Retractions trigger further retractions or corrections ("domino effect")

As noted above, retraction of one of the Surgisphere-linked large-scale registry studies [66] led to multiple corrections and further retractions[123, 127, 134, 135]. A review of effects of vitamin D deficiency in COVID-19 patients also required correction [136] to remove a citation of a retracted study [72]. Such chains of further retractions or corrections may create confusion as to which sources of information are reliable, unless the document histories are explicitly marked.
