# Supplementary material for: Inconsistent and incomplete retraction of published research: A cross-sectional study on Covid-19 retractions and recommendations to mitigate risks for research, policy and practice
Source: PLoS One. 2021 Oct 27;16(10):e0258935. doi: 10.1371/journal.pone.0258935 (PMC8550405; doi:10.1371/journal.pone.0258935)
Supplement: S2 Appendix — (PDF) [file pone.0258935.s002.pdf]

## S2 APPENDIX Medline search strategy

Ovid MEDLINE(R) and Epub Ahead of Print, In-Process & Other Non-Indexed Citations, Daily and Versions(R)

- Original search: 1946 to July 10, 2020
- Update search: 1946 to December 19, 2020

| | |
|---|---|
| 1 | retracted publication.pt. |
| 2 | retracted publication/ |
| 3 | "retraction of publication"/ |
| 4 | (retraction or retracted or withdrawn).ti. |
| 5 | 1 or 2 or 3 or 4 |
| 6 | (201912$ or 2020$).dt. |
| 7 | 5 and 6 |
| 8 | (exp coronavirus/ or coronavirus*.mp.) and (wuhan or beijing or shanghai or 2019-nCoV or nCoV or COVID-19 or SARS-CoV-2).mp. |
| 9 | coronavirus*.ti. or (novel coronavirus*.mp. and (exp china/ or china.mp.)) or ((pneumonia.mp. or exp pneumonia/) and Wuhan.mp.) |
| 10 | ("COVID-19" or "2019-nCoV" or "SARS-CoV-2").mp. or exp Coronavirus Infections/ |
| 11 | 9 or 10 |
| 12 | (201912$ or 2020$).dt. |
| 13 | 11 and 12 |

| | |
|---|---|
| 14 | 8 or 13 |
| 15 | 5 and 14 |
| 16 | limit 15 to dt=20200710-20201219 (update search only) |
