# Supplementary material for: Inconsistent and incomplete retraction of published research: A cross-sectional study on Covid-19 retractions and recommendations to mitigate risks for research, policy and practice
Source: PLoS One. 2021 Oct 27;16(10):e0258935. doi: 10.1371/journal.pone.0258935 (PMC8550405; doi:10.1371/journal.pone.0258935)
Supplement: S3 Appendix — (PDF) [file pone.0258935.s003.pdf]

S3 APPENDIX Characteristics of retracted COVID-19 articles (N=46) **(References are listed below the table)**

Reference	Version(s) available at source journal or preprint server at 05 September 2020	Retractor	Paper source (journal or preprint server)	Study type	Retraction reason	Comments
Adjodah et al. 2020 [1] (USA)	Retraction notice and full text with all pages clearly watermarked "Withdrawn" available at medRxiv preprint server (via Google Scholar)	Authors	medRxiv preprint server	Analysis of existing epidemiological datasets	Increased rates of SARS Cov-2 in the areas originally analysed; authors will provide new analyses to address this	Original unmarked abstract available in WHO COVID literature collection at 19th December 2020
Alhowary et al. 2020 [2] (Jordan)	Only the retraction notice is available at the journal website	Authors and/or journal	Annals of Medicine and Surgery	Case series	Not stated	Original unmarked full text available on ResearchGate at 19th December 2020
An et al. 2020 [3] (China)	Retraction notice and original unmarked abstract available at journal website. Abstract page of journal states a retraction has been published but access to the retraction notice and the full text article requires a subscription	Authors	Annals of Clinical and Laboratory Science (not open access)	Case series	Major error in data entry for analysis	Original unmarked abstract still available at journal website (see left) and on ResearchGate at 19th December 2020
Argyro-poulos et al. 2020 [4] (USA)	The retraction notice [5] and original article which was duplicated are available at the journal website	Journal	American Journal of Pathology	Retrospective analysis	Accidental duplicate publication	

Reference	Version(s) available at source journal or preprint server at 05 September 2020	Retractor	Paper source (journal or preprint server)	Study type	Retraction reason	Comments
Bae et al. 2020 [6] (S Korea)	Retraction notice [7] and marked full text available at journal website:<br><br>Original online full text with single one-line “retracted” statement above abstract<br><br>Original PDF full text with all pages watermarked “retracted”	Journal	Annals of Internal Medicine	Prospective controlled comparison	Data reporting error	
Beato-Vibora 2020 [8] (Spain)	Retraction notice and the original PDF full text, with “Retracted:” added to the article title available at the journal website [8]	Journal	Diabetes Technology & Therapeutics	Before-after study	Violation of publication ethics	Study reported in detail in Medscape Without indication of retraction at 19th December 2020. Note added in Medscape by 6th January 2021 that the cited study had been retracted.
Bility et al. 2020 [9] (USA)	Only the retraction notice [9] is available at the journal website	Authors and editors	Science of the Total Environment	Comparative/ correlative study of	Not stated	Original unmarked full text available on

Reference	Version(s) available at source journal or preprint server at 05 September 2020	Retractor	Paper source (journal or preprint server)	Study type	Retraction reason	Comments
				relationship of COVID-19 to geologic factors		ResearchGate at 19th December 2020
Cercy 2020 [10] (USA)	Original abstract, headed "withdrawn"<br><br>Original PDF full text, unmarked	Authors	Preprint server (medRxiv)	Medical record review	Privacy issues regarding study conduct (contested by author)	Original unmarked full text available on the preprint server (see left) and on ResearchGate at 19th December 2020
Chen et al. 2020 [11] (multi-national)	3 original versions of abstract, all headed "withdrawn" (v1, v2, v3)<br><br>3 original PDF versions of retracted full text, all unmarked (v1, v2, v3).	Authors	Preprint server (bioRxiv)	Prediction model	Lack of full author consent	Original unmarked full text available on ResearchGate at 19th December 2020
Chen et al. 2020 [12] (China)	Only the retraction notice [12] is available at the journal website	Authors	Liver International (published online as an accepted article)	Unclear - we do not have access to the full text.	Authors decided on reflection that the opinions expressed were incomplete [12]<br><br>NB original first author was Chen but subsequent	Journal site reports original article cited in two research papers [13, 14]

Reference	Version(s) available at source journal or preprint server at 05 September 2020	Retractor	Paper source (journal or preprint server)	Study type	Retraction reason	Comments
					correspondence refers to Zhou (corresponding author)	
Chu et al. 2020 [15] (multi-national)	Original abstract, headed "withdrawn"<br><br>Original PDF full text, all pages watermarked "withdrawn"<br><br>Original supplementary material, unmarked (file 1, file 2)	Authors	Preprint server (bioRxiv)	Molecular simulation study	To conduct further verification experiments	Original unmarked abstract available on ResearchGate at 19th December 2020
Davido et al. 2020 [16] (France)	Original abstract, headed "withdrawn"<br><br>Original PDF full text, unmarked	Authors	Preprint server (medRxiv)	Retrospective comparison	To revise after peer review following hydroxychloroquine controversy	Original unmarked full text available on the preprint server (see left) at 13th December 2020
Dumantepe et al. 2020 [17]	Only the retraction notice is available at the journal website	Authors	Heart, Lung and Circulation	Unknown. Full text not available	Not stated. A temporary removal notice on PMC states the reason for	Original unmarked abstract still available at

Reference	Version(s) available at source journal or preprint server at 05 September 2020	Retractor	Paper source (journal or preprint server)	Study type	Retraction reason	Comments
(Turkey)	(withdrawn article marked in press)				removal will be specified in the replacement article, or the article will be reinstated.	ClinOwl website at 19th December 2020
El Assaad et al. 2020 [18] (USA)	The retraction notice and the original article are available at the journal website	Journal	JACC Case Reports	Case study	Accidental duplicate publication	
Etemadifar et al. 2020 [19] (Iran)	Only the retraction notice is available at the journal website	Authors and Editor	Revue Neurologique (published online as an accepted article)	Unclear - we do not have access to the full text.	Not stated	Original unmarked abstract available online at Chinese website
Fioranelli et al. 2020 [20] (multi-national)	Only retraction notice is available at the journal website	Journal	Journal of Biological Regulators & Homeostatic Agents	Theoretical study/review badged as an editorial	Manipulation of the peer review<br><br>Science Integrity Digest blog (with archive of the original full text)	Original unmarked full text available from multiple online sites: 47 cites on 5th September 2020 and 60 sites on 19th December 2020 and 26th June 2021 as listed in Google Scholar (see Appendix 4).

Reference	Version(s) available at source journal or preprint server at 05 September 2020	Retractor	Paper source (journal or preprint server)	Study type	Retraction reason	Comments
						Original unmarked abstract available in WHO COVID literature collection and on ResearchGate at 19th December 2020
Gaibazzi et al. 2020 [21] (multi-national)	2 original versions of abstract, both headed "withdrawn" (v1, v2)<br><br>2 original PDF versions of retracted full text, both unmarked (v1, v2)<br><br>Original supplementary material, unmarked	Authors (as requested by their IRB)	Preprint server (medRxiv)	Retrospective case series	Objectives not approved by local IRB	Original full text available on the preprint server (see left) and ResearchGate as unretracted version at 19th December 2020
Gaibazzi et al. 2020 [22] (multi-national)	2 original versions of abstract, both headed "withdrawn" (v1, v2)<br><br>2 original PDF versions of retracted full text, both unmarked (v1, v2)	Authors	Preprint server (medRxiv)	Retrospective case series	Objectives not approved by local IRB	Study reported in detail by national newspaper [23]<br><br>Original full text available on the preprint server (see left) at 19th December 2020

Reference	Version(s) available at source journal or preprint server at 05 September 2020	Retractor	Paper source (journal or preprint server)	Study type	Retraction reason	Comments
Hedima et al. 2020 [24] (Nigeria)	Retraction notice [25] plus the original article which had been duplicated available at journal website	Journal	Research in Social and Administrative Pharmacy	Narrative review	Accidental duplicate publication	
Huang & Zhao 2020 [26] (China)	Full text available at journal website with all pages clearly watermarked "Retracted"<br><br>(duplicate of another paper [27])	Authors and Editor	Psychology, Health and Medicine	Web-based cross-sectional survey	Duplicate publication and failure of one author to satisfy authorship criteria [28] (October 2020)	Triple publication - same as Huang & Zhao [29]
Huang & Zhao 2020 [29] (China)	Full text available at journal website with all pages clearly watermarked "Retracted"<br><br>(duplicate of another paper [27])	Editor in Chief	Asian Journal of Psychiatry	As above – same study	Duplicate publication [30] (June 2020)	Triple publication – same as Huang & Zhao [26]
Irshad et al. [31] (Pakistan)	Only retraction notice [31] is available at the journal website	Journal	International Journal of Mental Health Nursing	Online cross-sectional survey	Reuse of a copyrighted questionnaire without permission	Original unmarked full text available on PubMed Central and

Reference	Version(s) available at source journal or preprint server at 05 September 2020	Retractor	Paper source (journal or preprint server)	Study type	Retraction reason	Comments
						original abstract available on ResearchGate at 19th December 2020
Kanwar 2020 [32] (USA)	Retraction notice only available on PubMed; no information at the journal website	Journal	American Journal of Respiratory & Critical Care Medicine	Unclear - full text not available	Not stated	Original unmarked abstract available on ClinOwl at 19th December 2020
Karami et al. 2020 [33] (Iran)	None	Authors	Travel Medicine and Infectious Disease	Case study	Failure to demonstrate first event	Original unmarked abstract available on ResearchGate at 19th December 2020
Kim et al. 2020 [34] (S Korea)	Original abstract, headed "withdrawn"<br><br>Original PDF full text, unmarked	Authors	Preprint server (medRxiv)	Retrospective cohort study	Due to "controversy about HCQ and potential changes to results after peer review"	Original unmarked full text available on the preprint server (see left) at 19th December 2020
Kumar 2020 [35] (India)	None	No information	Journal of Molecular Pharmaceuticals and Regulatory Affairs	No information	No information	Unclear

Reference	Version(s) available at source journal or preprint server at 05 September 2020	Retractor	Paper source (journal or preprint server)	Study type	Retraction reason	Comments
Luowei et al. 2020 [36] (China)	Original abstract [in Chinese], headed "withdrawn"	Journal	Practical Preventive Medicine	Case series	No information	Study was described in considerable full-page detail in national newspapers [37, 38]
Maltezou et al. 2020 [39] (Greece)	Only the retraction notice was available at the journal website at 19th December 2020; however, this had been removed and a version of the full text had been reinstated without any accompanying explanation by 6th January 2021	Not reported	Journal of Hospital Infection	Unknown. Abstract and full text not available	Not stated. A temporary removal notice was present on Pubmed at 19th December 2020 but had been removed by 6th January 2021.	The temporary removal notice stated the reason for removal would be specified in the replacement article, or the article would be reinstated, but no explanation was provided
Mehra et al. 2020 [40] (USA)	Original online full text with all pages watermarked "retracted"<br><br>Original PDF full text with all pages watermarked "retracted"	Authors (following journal Expression of concern [41])	Lancet	Multinational registry analysis	Data veracity concerns	Impact on WHO international hydroxy-chloroquine trial [42]

Reference	Version(s) available at source journal or preprint server at 05 September 2020	Retractor	Paper source (journal or preprint server)	Study type	Retraction reason	Comments
Mehra et al. 2020 [43] (USA)	Original online full text with narrow "retracted" banner on home page (article itself unmarked)<br><br>Original PDF full text with "retracted" statement at top of first page	Authors (following journal Expression of concern [44])	New England Journal of Medicine	Multinational registry analysis	Ditto, same study	Cited before retraction in NEJM editorial [45] which had not been altered at 19th December 2020<br><br>Temporarily cited by WHO guidelines [46]
Mulvey et al. 2020 [47] (USA)	Retraction notice plus the original article which had been duplicated available at journal website	Publisher	Annals of Diagnostic Pathology	Case series	Accidental duplication of an article already published in the same journal by the same authors (slightly different title)	Original unmarked abstract still available on ResearchGate at 19th December 2020
Nouvier et al. 2020 [48] (France)	Original PDF [in English and French], watermarked "retracted"	Authors	Bulletin de la Dialyse à Domicile	Case study	Suspected false positive result	
Parves et al. 2020 [49] (Bangla-desh)	Original abstract, headed "withdrawn"<br><br>Original PDF full text, all pages watermarked "withdrawn"	Authors	Preprint server (bioRxiv)	Molecular analysis	Lack of consent to use data	

Reference	Version(s) available at source journal or preprint server at 05 September 2020	Retractor	Paper source (journal or preprint server)	Study type	Retraction reason	Comments
Patel et al. 2020 [50] (USA)	None (NB: Original PDF of full text available from alternative online source, all pages watermarked "retracted")	Authors	Preprint server (SSRN)	Multinational registry analysis	No information	Controversial link to Surgisphere company implicated in research misconduct [51]
Patel et al. 2020 [52] (USA)	None	Authors	Preprint server (SSRN)	Multinational registry analysis	No information	Ditto [51]<br><br>Original unmarked full text available at alternative online source at 19th December 2020
Pradhan et al. 2020 [53] (India)	Original abstract, headed "withdrawn"<br><br>Original PDF full text, all pages watermarked "withdrawn"	Authors	Preprint server (bioRxiv)	Genomic analysis	To revise based on feedback	Original unmarked full text available on ResearchGate at 19th December 2020<br><br>Among the top 10 COVID-19 preprints tweeted up to 30th April 2020 [54]

Reference	Version(s) available at source journal or preprint server at 05 September 2020	Retractor	Paper source (journal or preprint server)	Study type	Retraction reason	Comments
Raharusuna et al. 2020 [55] (Indonesia)	None	No information	Preprint server (SSRN)	Retrospective cohort study	No information	Original PDF full text available, unmarked, at alternative online source at 19th December 2020
Singh et al. 2020 [56] (India)	Full text available at journal website with all pages clearly watermarked "Retracted"	Editorial Board and Publication Ethics Committee	Korean Journal of Anesthesiology	Brief narrative review	Plagiarism (September 2020)	
Siyu et al. 2020 [57] (China)	Original abstract, headed "withdrawn"<br><br>Original PDF full text, unmarked	Authors	Preprint server (medRxiv)	Online questionnaire survey	To conduct further verification experiments	Original unmarked full text available on the preprint server (see left) and ResearchGate at 19th December 2020
Vavougios 2020 [58] (Greece)	Only the retraction notice is available at the journal website	Authors and/or editor	Free Radical Biology and Medicine	Gene sequencing study	Not stated	Original unmarked abstract available on ResearchGate at 19th December 2020
Wang et al. 2020 [59] (China)	None	No information	Zhonghua Er Ke Za Zhi	Case series/registry analysis	No information	Original unmarked abstract available (in Chinese) on ResearchGate at 19th December 2020

Reference	Version(s) available at source journal or preprint server at 05 September 2020	Retractor	Paper source (journal or preprint server)	Study type	Retraction reason	Comments
Wang et al. 2020 [60] (multi-national)	Original PDF full text with all pages watermarked "retracted"	Authors	Cellular & Molecular Immunology	In vitro immunology study	Major flaw in methodology	
Wei et al. 2020 [61] (China)	Only the retraction notice is available at the journal website	Authors and/or editor	Clinical Imaging	Retrospective imaging case series analysis	Not stated but appears to be a duplicate of an article in Journal of X-Ray Science & Technology	Original unmarked abstract in Clinical Imaging journal available from ResearchGate at 19th December 2020
Yang et al. 2020 [62] (multi-national)	Original abstract, headed "withdrawn"<br><br>Original PDF full text, unmarked<br><br>Original supplemental material, unmarked	Authors	Preprint server (medRxiv)	Case registry analysis	To revise with more up to date data	Original unmarked full text available on the preprint server (see left) at 19th December 2020<br><br>Among the top 10 COVID-19 preprints cited up to 30th April 2020 [54]
Zhang et al. 2020 [63] (China)	Only the retraction notice is available at the journal website	Journal	International Journal of Nursing Studies	Cross-sectional survey	Not stated	Original unmarked abstract available on ResearchGate at 19th December 2020

Reference	Version(s) available at source journal or preprint server at 05 September 2020	Retractor	Paper source (journal or preprint server)	Study type	Retraction reason	Comments
Zhuang et al. 2020 [64] (China)	None	Journal<br><br>From the abstract/editor office's response: The article was decided to be offline by the editorial board from the pre-publish lists.	Chinese Journal of Epidemiology [in Chinese]	Test accuracy study	Conclusions were based on theory not field epidemiology data & thus further research needed.	Cited by the US White House Coronavirus Task Force. [65]

References (NB reference numbers in this appendix differ from those in the main paper)

1. Adjodah D, Dinakar K, Fraiberger SP, Rutherford GW, Glidden DV, Gandhi M. [WITHDRAWN] Decrease in Hospitalizations for COVID-19 after Mask Mandates in 1083 U.S. Counties. medRxiv Preprint Server. 2020.

2. Alhowary AA, Aleshawi AJ, Othman AR, Obeidat AY, Khader YS. WITHDRAWN: Mechanical ventilation in COVID-19: Is it due to patient or virology factors? Annals of Medicine and Surgery. Available online June 2020.

3. An X-S, Li X-Y, Shang F-T, Yang S-F, Zhao J-Y, Yang X-Z, et al. [RETRACTED] Clinical characteristics and blood test results in COVID-19 patients. . Annals of Clinical and Laboratory Science. 2020;50(3):299-307.

4. Argyropoulos KV, Serrano A, Hu J, Black M, Feng X, Shen G, et al. Association of Initial Viral Load in Severe Acute Respiratory Syndrome Coronavirus 2 (SARS-CoV-2) Patients with Outcome and Symptoms. American Journal of Pathology. 2020;190(9):1881-1887.

5. Argyropoulos KV, Serrano A, Hu J, Black M, Feng X, Shen G, et al. WITHDRAWN: Association of initial viral load in SARS-CoV-2 patients with outcome and symptoms. American Journal of Pathology. Available online July 2020.

6. Bae S, Kim MC, Kim JY, Cha HH, Lim JS, Jung J, et al. [RETRACTED] Effectiveness of Surgical and Cotton Masks in Blocking SARS-CoV-2: A Controlled Comparison in 4 Patients. Annals of Internal Medicine. 2020;173(1):W22-W23.

7. Bae S, Kim M-C, Kim JY, Cha H-H, Lim JS, Jung J, et al. Notice of Retraction: Effectiveness of Surgical and Cotton Masks in Blocking SARS-CoV-2. Annals of Internal Medicine. 2020; Available online June 2020.

8. Beato-Vibora PI. RETRACTED: No deleterious effect of lockdown due to COVID-19 pandemic on glycaemic control, measured by glucose monitoring, in adults with type 1 diabetes. Diabetes Technology and Therapeutics. Available online May 2020.

9. Bility MT, Agarwal Y, Ho S, Castronova I, Beatty C, Biradar S, et al. WITHDRAWN: Can Traditional Chinese Medicine provide insights into controlling the COVID-19 pandemic: Serpentinization-induced lithospheric long-wavelength magnetic anomalies in Proterozoic bedrocks in a weakened geomagnetic field mediate the aberrant transformation of biogenic molecules in COVID-19 via magnetic catalysis. Science of the Total Environment. Available online October 2020.

10. Cercy SP. [WITHDRAWN] Psychiatric predictors of COVID-19 outcomes in a skilled nursing facility cohort. medRxiv Preprint Server. 2020.

11. Chen Z, Zhang W, Lu Y, Guo C, Guo Z, Liao C, et al. [WITHDRAWN] From SARS-CoV to Wuhan 2019-nCoV: Will History Repeat Itself? bioRxiv Preprint Server. 2020.

12. Chen P, Lei J, Wy Y, Liu G, B Z. [WITHDRAWN] Liver impairment associated with disease progression in COVID 19 patients. Liver International. 2020; 40(9): 2308.

13. Portincasa P, Krawczyk M, Machill A, Lammert F, Di Ciaula A. Hepatic consequences of COVID-19 infection. Lapping or biting? European Journal of Internal Medicine. 2020;77:18-24.

14. Effenberger M, Grander C, Fritsche G, Bellmann-Weiler R, Hartig F, Wildner S, et al. Liver stiffness by transient elastography accompanies illness severity in COVID-19. BMJ Open Gastroenterology. 2020;7:e000445.

15. Chu P, Zhou Z, Gao Z, Cai R, Wu S, Sun Z, et al. [WITHDRAWN] Computational analysis suggests putative intermediate animal hosts of the SARS-CoV-2. bioRxiv Preprint Server. 2020.

16. Davido B, Lansaman T, Bessis S, Lawrence C, Alvarez J-C, Mascitti H, et al. [WITHDRAWN] Hydroxychloroquine plus azithromycin: a potential interest in reducing in-hospital morbidity due to COVID-19 pneumonia (HI-ZY-COVID)? medRxiv Preprint Server. 2020.

17. Dumantepe M, Aydin S, Yildiz E, Okur HK, Kocagoz AS, Gundogdu Y, et al. WITHDRAWN: Subsegmental Thrombus in COVID-19 Pneumonia: Immuno-Thrombosis or Pulmonary Embolism? Data Analysis of Hospitalized Patients with Coronavirus Disease. Heart, Lung & Circulation. 2020;24:24.

18. El-Assaad I, Hood-Pishchany MI, Kheir J, Mistry K, Dixit A, Halyabar O, et al. Complete Heart Block, Severe Ventricular Dysfunction, and Myocardial Inflammation in a Child With COVID-19 Infection. JACC: Case Reports. 2020;2(9):1351-1355.

19. Etemadifar M, Aghababaee A, Sedaghat N, Rayani M, Nouri H, Abhari A, et al. WITHDRAWN: Incidence and mortality of COVID-19 in Iranian multiple sclerosis patients treated with disease-modifying therapies. Revue Neurologique. 2020;Online ahead of print.

20. Fioranelli M, Sepheri A, Roccia MG, Jafferani M, Olisova OY, Lomonosov KM, et al. RETRACTED: 5G Technology and induction of coronavirus in skin cells. Journal of Biological Regulators and Homeostatic Agents. 2020;34(4): available online July 2020.

21. Gaibazzi N, Martini C, Mattioli M, Tuttolomondo D, Guidorrossi A, Suma S, et al. [WITHDRAWN] Lung disease severity, coronary artery calcium, coronary inflammation and mortality in Coronavirus disease 2019. medRxiv Preprint Server. 2020.

22. Gaibazzi N, Tuttolomondo D, Guidorossi A, Botti A, Tedeschi A, Martini C, et al. [WITHDRAWN] Smoking Prevalence is Low in Symptomatic Patients Admitted for COVID-19. medRxiv Preprint Server. 2020.

23. Chalmers V. MORE evidence emerges that smokers are protected from coronavirus: Italian study finds them FIVE TIMES less likely to end up in hospital (but almost twice as likely to die if they do). Daily Mail (Mail Online). 2020 11th May.

24. Hedima EW, Adeyemi MS, Ikunaiye NY. Community Pharmacists: On the frontline of health service against COVID-19 in LMICs. Research in Social and Administrative Pharmacy. 2021;17(1):1964-1966.

25. Hedima EW, Adeyemi MS, Ikunaiye NY. WITHDRAWN: Community pharmacists: On the frontline of health service against COVID-19 in LMICs. Research in Social and Administrative Pharmacy. Available online April 2020.

26. Huang Y, Zhao N. [RETRACTED] Mental health burden for the public affected by the COVID-19 outbreak in China: Who will be the high-risk group? Psychology, Health & Medicine. 2020;26(1):23-24.

27. Huang Y, Zhao N. Generalized anxiety disorder, depressive symptoms and sleep quality during COVID-19 outbreak in China: a web-based cross-sectional survey. Psychiatry Research. 2020;288:112954.

28. Editor and Publisher of Psychology Health & Medicine. Retraction: Mental health burden for the public affected by the COVID-19 outbreak in China: Who will be the high-risk group? Psychology, Health & Medicine. 2021; 26(5):656.

29. Huang Y, Zhao N. RETRACTED: Chinese mental health burden during the COVID-19 pandemic. Asian Journal of Psychiatry. 2020;51: 102052:1-3.

30. Asian Journal of Psychiatry. RETRACTED: Chinese mental health burden during the COVID-19 pandemic. Asian Journal of Psychiatry. 2020;51:102052:1.

31. Editor and Publisher of International Journal of Mental Health Nursing. Withdrawn: How perceived threat of Covid-19 causes turnover intention among Pakistani nurses: A moderation and mediation analysis. International Journal of Mental Health Nursing. Available online August 2020.

32. Kanwar BA. WITHDRAWN: Proposal for Initiative of Evidence-based Treatment of COVID-19 Patients with Worsening Hypoxia. American Journal of Respiratory and Critical Care Medicine. Available October 2020.

33. Karami P, Naghavi M, Feyzi A, Aghamohammadi M, Novin MS, Mobaien A, et al. WITHDRAWN: Mortality of a pregnant patient diagnosed with COVID-19: A case report with clinical, radiological, and histopathological findings. Travel Medicine and Infectious Disease. 2020:101665.

34. Kim MS, Jang S-W, Park Y-K, Kim B-o, Hwang T-H, Kang SH, et al. [WITHDRAWN] Treatment response to hydroxychloroquine, lopinavir/ritonavir, and antibiotics for moderate COVID 19: A first report on the pharmacological outcomes from South Korea. medRxiv Preprint Server. 2020.

35. Kumar D. [RETRACTED] Corona Virus Killed by Sound Vibrations Produced by Thali or Ghanti: A Potential Hypothesis. Journal of Molecular Pharmaceuticals and Regulatory Affairs. 2020;2(2).

36. Luowei L, Zheng H, Shanliang X, Hao Y, Xinping J, Hui W, et al. [A new coronal form of aerosol transmission in public transportation Epidemiological investigation of viral pneumonia cluster epidemic situation.] [Chinese] Practical Preventive Medicine. 2020;1-3.

37. Chalmers V. How one man spread coronavirus to NINE other people on his bus in China: Scientists find the deadly virus can travel four times the 'safe' distance and linger in the air for over 30 minutes in confined places. Daily Mail (Mail Online). 2020 11th March.

38. Chen S. Coronavirus can travel twice as far as official ‘safe distance’ and stay in air for 30 minutes, Chinese study finds. South China Morning Post. 2020 9th March.

39. Maltezou HC, Dedoukou X, Tsonou P, Tseroni M, Raftopoulos V, Pavli A, et al. TEMPORARY REMOVAL: Hospital factors associated with SARS-CoV-2 infection among healthcare personnel in Greece. Journal of Hospital Infection. 2020;109:40-43.

40. Mehra MR, Desai SS, Ruschitzka F, Patel AN. RETRACTED: Hydroxychloroquine or chloroquine with or without a macrolide for treatment of COVID-19: a multinational registry analysis. Lancet. Available May 2020.

41. The Lancet Editors. Expression of concern: Hydroxychloroquine or chloroquine with or without a macrolide for treatment of COVID-19: a multinational registry analysis. Lancet. 2020;395(10240):e102.

42. Lovelace B, Feuer W. World Health Organization resumes coronavirus trial on malaria drug hydroxychloroquine after examining safety concerns. CNBC. 2020 3rd June.

43. Mehra MR, Desai SS, Kuy S, Henry TD, Patel AN. Retraction: Cardiovascular Disease, Drug Therapy, and Mortality in Covid-19. N Engl J Med. DOI: 10.1056/NEJMoa2007621. New England Journal of Medicine. 2020;382(26):2582.

44. Rubin EJ. Expression of Concern: Mehra MR et al. Cardiovascular Disease, Drug Therapy, and Mortality in Covid-19. N Engl J Med. DOI: 10.1056/NEJMoa2007621. NEJM. 2020;382:2464:1.

45. Jarcho JA, Ingelfinger JR, Hamel MB, D'Agostino RB, Harrington DP. Inhibitors of the renin–angiotensin–aldosterone system and Covid-19. NEJM. 2020;382:2462-2464.

46. WHO (World Health Organisation). Smoking and COVID-19. Scientific brief 30 June 2020.

47. Mulvey JJ, Magro CM, Ma LX, Nuovo GJ, Baergen RN. WITHDRAWN: A mechanistic analysis placental intravascular thrombus formation in COVID-19 patients. Annals of Diagnostic Pathology. 2020;46: 151529.

48. Nouvier M, Chalencon E, Novle-Catin E, Pelletier S, Hallonet P, Charre C, et al. [RETRACTED] First viral replication of Covid-19 identified in the peritoneal dialysis fluid of a symptomatic patient. Bulletin de la Dialyse à Domicile. 2020;3(1):54503.

49. Parves MR, Riza YM, Mahmud S, Islam R, Ahmed S, Evy BA, et al. [WITHDRAWN] Analysis of Ten Microsecond simulation data of SARS-CoV-2 dimeric main protease. bioRxiv Preprint Server. 2020.

50. Patel A, Desai S. [RETRACTED] Ivermectin in COVID-19 Related Critical Illness. SSRN Preprint Server. 2020.

51. Offord C. Surgisphere Sows Confusion About Another Unproven COVID-19 Drug. TheScientist. 2020 16th June.

52. Patel AN, Desai SS, Grainger DW, Mehra MR. [RETRACTED] Usefulness of ivermectin in Covid-19 illness. Online source. 2020. https://kitasato-infection-control.info/swfu/d/COVID-19_Illness.pdf

53. Pradhan P, Pandey AK, Mishra A, Gupta P, Tripathi PK, Menon MB, et al. [WITHDRAWN] Uncanny similarity of unique inserts in the 2019-nCoV spike protein to HIV-1 gp120 and Gag. bioRxiv Preprint Server. 2020.

54. Fraser N, Brierley L, Dey G, Polka JK, Pálfy M, Nanni F, et al. Preprinting the COVID-19 pandemic [preprint pending peer review]. bioRxiv preprint server. 2020:1-34.

55. Raharusuna P, Priambada S, Budiarti C, Agung E, Budi C. [RETRACTED] Patterns of COVID-19 Mortality and Vitamin D: An Indonesian Study. SSRN Preprint server. 2020.

56. Singh A. [RETRACTED] Noninvasive versus invasive ventilation: One modality cannot fit all during COVID-19 outbreak. Korean Journal of Anesthesiology. 2020;73(4):359-361.

57. Siyu C, Xia M, Wen W, Cui L, Yang W, Liu S, et al. [WITHDRAWN] Mental health status and coping strategy of medical workers in China during The COVID-19 outbreak. medRxiv Preprint Server. 2020.

58. Vavougios GD. WITHDRAWN: Selenium - associated gene signatures within the SARS-CoV-2 - host genomic interaction interface. Free Radical Biology & Medicine. Available online July 2020.

59. Wang XF, Yuan J, Zheng YJ, Chen J, Bao YM, Wang YR, et al. [Retracted: Clinical and epidemiological characteristics of 34 children with 2019 novel coronavirus infection in Shenzhen]. Zhonghua Er Ke Za Zhi. 2020;58:E008.

60. Wang X, Xu W, Hu G, Xia S, Sun Z, Liu Z, et al. RETRACTED ARTICLE: SARS-CoV-2 infects T lymphocytes through its spike protein-mediated membrane fusion. Cellular & Molecular Immunology. Available online April 2020.

61. Wei J, Lei P, Yang H, Fan B, Qiu Y, Zeng B, et al. WITHDRAWN: Analysis of thin-section CT in patients with coronavirus disease (COVID-19) after hospital discharge. Clinical Imaging. Available online May 2020.

62. Yang Y, Lu Q, Liu M, Wang Y, Zhang A, Jalali N, et al. [WITHDRAWN] Epidemiological and clinical features of the 2019 novel coronavirus outbreak in China. medRxiv Preprint Server. 2020.

63. Zhang X, Jiang Z, Yuan X, Wang Y, Huang D, Hu R, et al. WITHDRAWN: Nurses reports of actual work hours and preferred work hours per shift among frontline nurses during coronavirus disease 2019 (COVID-19) epidemic: A cross-sectional survey. International Journal of Nursing Studies. 2020:103635.

64. Zhuang GH, Shen MW, Zeng LX, Mi BB, Chen FY, Liu WJ, et al. [WITHDRAWN: Potential false-positive rate among the 'asymptomatic infected individuals' in close contacts of COVID-19 patients]. Zhonghua Liu Xing Bing Xue Za Zhi. 2020;41(4):485-488.

65. Harris R. In Defense Of Coronavirus Testing Strategy, Administration Cited Retracted Study. NPR (National Public Radio). 2020 26th March.
