# Supplementary material for: Inconsistent and incomplete retraction of published research: A cross-sectional study on Covid-19 retractions and recommendations to mitigate risks for research, policy and practice
Source: PLoS One. 2021 Oct 27;16(10):e0258935. doi: 10.1371/journal.pone.0258935 (PMC8550405; doi:10.1371/journal.pone.0258935)
Supplement: S4 Appendix — (PDF) [file pone.0258935.s004.pdf]

S4 APPENDIX Timing of publication and retraction for papers published on preprint servers

(a) Timing of publication and retraction for papers published on preprint servers (bioRxiv, medRxiv, SSRN) and in academic journals (based on data available at 19th December 2020)

Paper	Publication date (2020)	Retraction date (2020)	Time to retraction	Source	Journal publisher
Adjodah et al. [1]	23 Oct	4 Nov	12 days	medRxiv	-
Alhowary et al. [2]	14 Jun	not reported [a]	unclear	Journal	Elsevier
An et al. [3]	not reported	not reported	unclear	Journal	Elsevier
Argyropoulos et al. [4]	7 Jul	not reported [a]	unclear	Journal	Elsevier
Bae et al. [5]	6 Apr	2 Jun	57 days	Journal	ACP Journals
Beato-Vibora [6]	12 May	27 July	76 days	Journal	Mary Ann Liebert
Bility et al. [7]	8 Oct	5 Nov [a] ("temporary removal")	28 days	Journal	Elsevier
Cercy [8]	26 May	21 Jun	26 days	medRxiv	-
Chen et al. [9]	27 Jan	28 Jan	1 day	bioRxiv	-
Chen et al. [10]	15 Apr	unclear (Sep issue of journal)	unclear	Journal	Wiley
Chu et al. [11]	5 Apr	15 Apr	10 days	bioRxiv	-
Davido et al. [12]	11 May	20 May	9 days	medRxiv	-
Dumantepe et al. [13]	24 Aug	not reported	unclear	Journal	Elsevier
El Assaad et al. [14]	27 May	not reported	unclear	Journal	Elsevier
Etemadifar et al. [15]	15 Sep	8 Oct [a]	23 days	Journal	Elsevier
Fioranelli et al. [16]	16 Jul	24 Jul [a]	8 days	Journal	Biolife
Gaibazzi et al. [17]	17 May	20 Jun	34 days	medRxiv	-
Gaibazzi et al. [18]	15 May	13 Jun	29 days	medRxiv	-
Hedima et al. [19]	19 Apr	not reported [a]	unclear	Journal	Elsevier
Huang & Zhao [20]	14 Apr	23 Oct [a]	192 days	Journal	Taylor & Francis
Huang & Zhao [21]	14 Apr	17 Nov [b]	217 days	Journal	Elsevier
Irshad et al. [22]	10 Aug	not reported	unclear	Journal	Wiley
Kanwar [23]	7 Oct	not reported [a]	unclear	Journal	ATS Journals
Karami et al. [24]	11 Apr	2 May [a]	21 days	Journal	Elsevier
Kim et al. [25]	18 May	14 Jun	27 days	medRxiv	-
Kumar [26]	Mar/Aug	not reported	unknown	Journal	MAT Journals
Luowei et al. [27]	early Mar [a]	mid Apr [a]	unclear	Journal	[Chinese]

Paper	Publication date (2020)	Retraction date (2020)	Time to retraction	Source	Journal publisher
Maltezou et al. [28]	22 Oct	Unclear ("temporary removal") [c]	unclear	Journal	Healthcare Infection Society
Mehra et al. [29]	22 May	5 Jun	14 days	Journal	Elsevier
Mehra et al. [30]	1 May	4 Jun	34 days	Journal	Massachusetts Medical Society
Mulvey et al. [31]	25 Apr	22 Jun [a]	58 days	Journal	Elsevier
Nouvier et al. [32]	13 Apr	20 Apr [a]	7 days	Journal	RDPLF
Parves et al. [33]	12 Apr	16 Apr	4 days	bioRxiv	-
Patel et al. [34]	"early Apr" [a]	"end of May" [a]	unclear	SSRN	-
Patel et al. [35]	not reported	not reported	unknown	SSRN	-
Pradhan et al. [36]	31 Jan	2 Feb	2 days	bioRxiv	-
Raharusuna et al. [37]	not reported	not reported	unknown	SSRN	-
Singh et al. [38]	8 Jul	14 Sep	68 days	Journal	Korean Society of Anesthesiologists
Siyu et al. [39]	25 Feb	7 Mar	11 days	medRxiv	-
Vavougios [40]	15 Jul	not reported	unclear	Journal	Elsevier
Wang [41]	unclear	not reported	unclear	Journal	[Chinese]
Wang et al. [42]	7 Apr	10 Jul	94 days	Journal	Nature Publishing Group
Wei et al. [43]	15 May	not reported	unclear	Journal	Elsevier
Yang et al. [44]	11 Feb	21 Feb	10 days	medRxiv	-
Zhang et al. [45]	16 May	not reported [a]	unclear	Journal	Elsevier
Zhuang et al. [46]	5 Mar	"a few days later" [a]	unclear	Journal	[Chinese]
[a] from Retraction Watch [b] from Pubmed [c] article reinstated by 6th January 2021 (see Appendix 2)					

S4 APPENDIX (b) Summary of time (days) from publication to retraction

Summary statistics	Preprint servers				Journals (n=31)
	bioRxiv (n=4)	medRxiv (n=8)	SSRN (n=3)	Total (n=15)	
**Number of papers with available data**	4	8	0	12	14
**Median**	3	19	no data	10.5	45.5
**Range**	1-10	9-34	no data	1 to 34	7-217
**Interquartile range**	1.75 to 5.5	10.75 to 27.5	no data	7.75 to 26.25	21.5 to 74

**Missing data (retraction and/or publication date not reported**	0	0	3	3	17

S4 APPENDIX (c) Publishers of the 31 articles that were retracted from scientific journals

Journal publisher	Total number of retracted articles (n=31)	Articles with unclear reason for retraction (n=12)	Articles with unclear date of retraction (n=17)
Elsevier	15 (48%)	7 [2, 7, 13, 15, 40, 43, 45]	9 [2-4, 13, 14, 19, 40, 43, 45]
Chinese publishers [a]	3 (7%)	2 [27, 41]	3 [27, 41, 46]
Wiley	2	0	2 [10, 47]
ACP Journals	1	0	
ATS Journals	1	1 [23]	1 [23]
Biolife	1	0	
Hospital Infection Society	1	1 [28]	1 [28]
Korean Society of Anaesthesiologists	1	0	
Mary Ann Liebert	1	0	
Massachusetts Medical Society (NEJM)	1	0	
MAT Journals	1	1 [26]	1 [26]
Nature publishing Group	1	0	
RDPLF	1	0	
Taylor & Francis	1	0	
[a] Three different publishers, which we have not identified separately here, whose journals publish primarily in the Chinese language			

References (NB reference numbers in this appendix differ from those in the main paper)

1. Adjodah D, Dinakar K, Fraiberger SP, Rutherford GW, Glidden DV, Gandhi M. [WITHDRAWN] Decrease in Hospitalizations for COVID-19 after Mask Mandates in 1083 U.S. Counties. medRxiv Preprint Server. 2020.

2. Alhowary AA, Aleshawi AJ, Othman AR, Obeidat AY, Khader YS. WITHDRAWN: Mechanical ventilation in COVID-19: Is it due to patient or virology factors? Annals of Medicine and Surgery. Available online June 2020.

3. An X-S, Li X-Y, Shang F-T, Yang S-F, Zhao J-Y, Yang X-Z, et al. [RETRACTED] Clinical characteristics and blood test results in COVID-19 patients. . Annals of Clinical and Laboratory Science. 2020;50(3):299-307.

4. Argyropoulos KV, Serrano A, Hu J, Black M, Feng X, Shen G, et al. WITHDRAWN: Association of initial viral load in SARS-CoV-2 patients with outcome and symptoms. American Journal of Pathology. Available online July 2020.

5. Bae S, Kim MC, Kim JY, Cha HH, Lim JS, Jung J, et al. [RETRACTED] Effectiveness of Surgical and Cotton Masks in Blocking SARS-CoV-2: A Controlled Comparison in 4 Patients. Annals of Internal Medicine. 2020;173(1):W22-W23.

6. Beato-Vibora PI. RETRACTED: No deleterious effect of lockdown due to COVID-19 pandemic on glycaemic control, measured by glucose monitoring, in adults with type 1 diabetes. Diabetes Technology and Therapeutics. Available online May 2020.

7. Bility MT, Agarwal Y, Ho S, Castronova I, Beatty C, Biradar S, et al. WITHDRAWN: Can Traditional Chinese Medicine provide insights into controlling the COVID-19 pandemic: Serpentinization-induced lithospheric long-wavelength magnetic anomalies in Proterozoic bedrocks in a weakened geomagnetic field mediate the aberrant transformation of biogenic molecules in COVID-19 via magnetic catalysis. Science of the Total Environment. Available online October 2020.

8. Cercy SP. [WITHDRAWN] Psychiatric predictors of COVID-19 outcomes in a skilled nursing facility cohort. medRxiv Preprint Server. 2020.

9. Chen Z, Zhang W, Lu Y, Guo C, Guo Z, Liao C, et al. [WITHDRAWN] From SARS-CoV to Wuhan 2019-nCoV: Will History Repeat Itself? bioRxiv Preprint Server. 2020:2020.2001.2024.919241.

10. Chen P, Lei J, Wy Y, Liu G, B Z. [WITHDRAWN] Liver impairment associated with disease progression in COVID 19 patients. Liver International. 2020; 40(9):2308.

11. Chu P, Zhou Z, Gao Z, Cai R, Wu S, Sun Z, et al. [WITHDRAWN] Computational analysis suggests putative intermediate animal hosts of the SARS-CoV-2. bioRxiv Preprint Server. 2020.

12. Davido B, Lansaman T, Bessis S, Lawrence C, Alvarez J-C, Mascitti H, et al. [WITHDRAWN] Hydroxychloroquine plus azithromycin: a potential interest in reducing in-hospital morbidity due to COVID-19 pneumonia (HI-ZY-COVID)? medRxiv Preprint Server. 2020.

13. Dumantepe M, Aydin S, Yildiz E, Okur HK, Kocagoz AS, Gundogdu Y, et al. WITHDRAWN: Subsegmental Thrombus in COVID-19 Pneumonia: Immuno-Thrombosis or Pulmonary Embolism? Data Analysis of Hospitalized Patients with Coronavirus Disease. Heart, Lung & Circulation. 2020;24:24.

14. El-Assaad I, Hood-Pishchany MI, Kheir J, Mistry K, Dixit A, Halyabar O, et al. Complete Heart Block, Severe Ventricular Dysfunction, and Myocardial Inflammation in a Child With COVID-19 Infection. JACC: Case Reports. 2020;2(9):1351-1355.

15. Etemadifar M, Aghababaee A, Sedaghat N, Rayani M, Nouri H, Abhari A, et al. WITHDRAWN: Incidence and mortality of COVID-19 in Iranian multiple sclerosis patients treated with disease-modifying therapies. Revue Neurologique. 2020;Online ahead of print.

16. Fioranelli M, Sepheri A, Roccia MG, Jafferani M, Olisova OY, Lomonosov KM, et al. RETRACTED: 5G Technology and induction of coronavirus in skin cells. Journal of Biological Regulators and Homeostatic Agents. 2020;34(4):Epub ahead of print.

17. Gaibazzi N, Martini C, Mattioli M, Tuttolomondo D, Guidorrossi A, Suma S, et al. [WITHDRAWN] Lung disease severity, coronary artery calcium, coronary inflammation and mortality in Coronavirus disease 2019. medRxiv Preprint Server. 2020.

18. Gaibazzi N, Tuttolomondo D, Guidorossi A, Botti A, Tedeschi A, Martini C, et al. [WITHDRAWN] Smoking Prevalence is Low in Symptomatic Patients Admitted for COVID-19. medRxiv Preprint Server. 2020.

19. Hedima EW, Adeyemi MS, Ikunaiye NY. WITHDRAWN: Community pharmacists: On the frontline of health service against COVID-19 in LMICs. Research in Social and Administrative Pharmacy. 2021;17(1):1964-1966.

20. Huang Y, Zhao N. [RETRACTED] Mental health burden for the public affected by the COVID-19 outbreak in China: Who will be the high-risk group? Psychology, Health & Medicine. 2020;26(1):23-24.

21. Huang Y, Zhao N. RETRACTED: Chinese mental health burden during the COVID-19 pandemic. Asian Journal of Psychiatry. 2020;51: 102052:1-3.

22. Irshad M, Khattak SA, Hassan MM, Majeed M, Bashir S. WITHDRAWN: How perceived threat of Covid-19 causes turnover intention among Pakistani nurses: A moderation and mediation analysis. International Journal of Mental Health Nursing. 2020;30(1):350-350.

23. Kanwar BA. WITHDRAWN: Proposal for Initiative of Evidence-based Treatment of COVID-19 Patients with Worsening Hypoxia. American Journal of Respiratory and Critical Care Medicine. 2020;Online ahead of print.

24. Karami P, Naghavi M, Feyzi A, Aghamohammadi M, Novin MS, Mobaien A, et al. WITHDRAWN: Mortality of a pregnant patient diagnosed with COVID-19: A case report with clinical, radiological, and histopathological findings. Travel Medicine and Infectious Disease. 2020:101665.

25. Kim MS, Jang S-W, Park Y-K, Kim B-o, Hwang T-H, Kang SH, et al. [WITHDRAWN] Treatment response to hydroxychloroquine, lopinavir/ritonavir, and antibiotics for moderate COVID 19: A first report on the pharmacological outcomes from South Korea. medRxiv Preprint Server. 2020.

26. Kumar D. [RETRACTED] Corona Virus Killed by Sound Vibrations Produced by Thali or Ghanti: A Potential Hypothesis. Journal of Molecular Pharmaceuticals and Regulatory Affairs. 2020;2(2).

27. Luowei L, Zheng H, Shanliang X, Hao Y, Xinping J, Hui W, et al. [A new coronal form of aerosol transmission in public transportation Epidemiological investigation of viral pneumonia cluster epidemic situation.] [Chinese] Practical Preventive Medicine. 2020;1-3.

28. Maltezou HC, Dedoukou X, Tsonou P, Tseroni M, Raftopoulos V, Pavli A, et al. TEMPORARY REMOVAL: Hospital factors associated with SARS-CoV-2 infection among healthcare personnel in Greece. Journal of Hospital Infection. 2020;109:40-43.

29. Mehra MR, Desai SS, Ruschitzka F, Patel AN. RETRACTED: Hydroxychloroquine or chloroquine with or without a macrolide for treatment of COVID-19: a multinational registry analysis. Lancet. Available online May 2020).

30. Mehra MR, Desai SS, Kuy S, Henry TD, Patel AN. Retraction: Cardiovascular Disease, Drug Therapy, and Mortality in Covid-19. N Engl J Med. DOI: 10.1056/NEJMoa2007621. New England Journal of Medicine. 2020;382(26):2582.

31. Mulvey JJ, Magro CM, Ma LX, Nuovo GJ, Baergen RN. WITHDRAWN: A mechanistic analysis placental intravascular thrombus formation in COVID-19 patients. Annals of Diagnostic Pathology. 2020;46: 151529.

32. Nouvier M, Chalencon E, Novle-Catin E, Pelletier S, Hallonet P, Charre C, et al. [RETRACTED] First viral replication of Covid-19 identified in the peritoneal dialysis fluid of a symptomatic patient. Bulletin de la Dialyse à Domicile. 2020;3(1):54503.

33. Parves MR, Riza YM, Mahmud S, Islam R, Ahmed S, Evy BA, et al. [WITHDRAWN] Analysis of Ten Microsecond simulation data of SARS-CoV-2 dimeric main protease. bioRxiv Preprint Server. 2020.

34. Patel A, Desai S. [RETRACTED] Ivermectin in COVID-19 Related Critical Illness. SSRN Preprint Server. 2020.

35. Patel AN, Desai SS, Grainger DW, Mehra MR. [RETRACTED] Usefulness of ivermectin in Covid-19 illness. Online source. 2020.

36. Pradhan P, Pandey AK, Mishra A, Gupta P, Tripathi PK, Menon MB, et al. [WITHDRAWN] Uncanny similarity of unique inserts in the 2019-nCoV spike protein to HIV-1 gp120 and Gag. bioRxiv Preprint Server. 2020.

37. Raharusuna P, Priambada S, Budiarti C, Agung E, Budi C. [RETRACTED] Patterns of COVID-19 Mortality and Vitamin D: An Indonesian Study. SSRN Preprint server. 2020.

38. Singh A. [RETRACTED] Noninvasive versus invasive ventilation: One modality cannot fit all during COVID-19 outbreak. Korean Journal of Anesthesiology. 2020;73(4):359-361.

39. Siyu C, Xia M, Wen W, Cui L, Yang W, Liu S, et al. [WITHDRAWN] Mental health status and coping strategy of medical workers in China during The COVID-19 outbreak. medRxiv Preprint Server. 2020.

40. Vavougios GD. WITHDRAWN: Selenium - associated gene signatures within the SARS-CoV-2 - host genomic interaction interface. Free Radical Biology & Medicine. Available online July 2020.

41. Wang XF, Yuan J, Zheng YJ, Chen J, Bao YM, Wang YR, et al. [Retracted: Clinical and epidemiological characteristics of 34 children with 2019 novel coronavirus infection in Shenzhen]. Zhonghua Er Ke Za Zhi. 2020;58:E008.

42. Wang X, Xu W, Hu G, Xia S, Sun Z, Liu Z, et al. RETRACTED ARTICLE: SARS-CoV-2 infects T lymphocytes through its spike protein-mediated membrane fusion. Cellular & Molecular Immunology. Available online April 2020.

43. Wei J, Lei P, Yang H, Fan B, Qiu Y, Zeng B, et al. WITHDRAWN: Analysis of thin-section CT in patients with coronavirus disease (COVID-19) after hospital discharge. Clinical Imaging. Available online May 2020.

44. Yang Y, Lu Q, Liu M, Wang Y, Zhang A, Jalali N, et al. [WITHDRAWN] Epidemiological and clinical features of the 2019 novel coronavirus outbreak in China. medRxiv Preprint Server. 2020.

45. Zhang X, Jiang Z, Yuan X, Wang Y, Huang D, Hu R, et al. WITHDRAWN: Nurses reports of actual work hours and preferred work hours per shift among frontline nurses during coronavirus disease 2019 (COVID-19) epidemic: A cross-sectional survey. International Journal of Nursing Studies. 2020:103635.

46. Zhuang GH, Shen MW, Zeng LX, Mi BB, Chen FY, Liu WJ, et al. [WITHDRAWN: Potential false-positive rate among the 'asymptomatic infected individuals' in close contacts of COVID-19 patients]. Zhonghua Liu Xing Bing Xue Za Zhi. 2020;41(4):485-488.

47. Editor and Publisher of International Journal of Mental Health Nursing. Withdrawn: How perceived threat of Covid-19 causes turnover intention among Pakistani nurses: A moderation and mediation analysis. International Journal of Mental Health Nursing. Available online August 2020.
