# Supplementary material for: Inconsistent and incomplete retraction of published research: A cross-sectional study on Covid-19 retractions and recommendations to mitigate risks for research, policy and practice
Source: PLoS One. 2021 Oct 27;16(10):e0258935. doi: 10.1371/journal.pone.0258935 (PMC8550405; doi:10.1371/journal.pone.0258935)
Supplement: S5 Appendix — (PDF) [file pone.0258935.s005.pdf]

S5 APPENDIX Sources of unmarked copies of the retracted paper by Fioranelli et al. 2020 (retracted on 24th July 2020)

Fioranelli M, Sepehri A, Roccia MG, Jafferani M, Olisova OY, Lemonosov KM, et al. RETRACTED: 5G Technology and induction of coronavirus in skin cells. Journal of Biological Regulators and Homeostatic Agents. Publication date 16th July 2020.

Google Scholar searches for "5G Technology and induction of coronavirus in skin cells" gave the following results:

5th September 2020: PDF available from 47 different sites

19th December 2020: PDF available from 71 different sites. Of these:

- 4 provided an unmarked original abstract only
- 1 was flagged as a malicious website and not checked
- 6 provided full text marked retracted
- 60 provided original unmarked full text – all links below gave access on 19th December 2020 and when checked again on 6th January 2021.
- On re-checking at 26 June 2021 three of the full-text links were no longer available but three new links had become available, as follows:

**Full text sources available on 19th December 2020 and remaining available on 26th June 2021**	
1	https://www.theothersideofmidnight.com/wp-content/uploads/2020/07/NIH-16th-July-2020-5G-Technology-and-induction-of-coronavirus-in-skin-cells-.pdf
2	http://medintegra.es/wp-content/uploads/2020/07/COVID-y-5g.pdf
3	https://niburu.co/images/2020/jul/5g.pdf
4	https://www.thelastamericanvagabond.com/wp-content/uploads/2020/07/FIORANELLI.pdf
5	http://mreengenharia.com.br/pdf/FIORANELLI.pdf
6	https://www.startlinken.nl/documents/FIORANELLI.pdf
7	https://ortodoxinfo.ro/wp-content/uploads/2020/07/5G_Coronavirus.pdf
8	https://frchan.bet/.media/ad285aecc1927ae76382d7562553ac1bf245f98c74b1605be49676c20a55c7a6.pdf
9	http://www.factwrecker.com/pdf-pages/EMF/FIORANELLI-Coronavirus-5g-EMF.pdf

10	https://www.mentealternativa.com/ma_media/2020/08/5G-Technology-and-induction-of-coronavirus-in-skin-cells.pdf (PDF available after bot filter security check step)
11	https://www.mentealternativa.com/wp-content/uploads/2020/08/5G-Technology-and-induction-of-coronavirus-in-skin-cells.pdf
12	http://www.partitoviola.it/docs/5G-Technology-and-induction-of-coronavirus-in-skin-cells-M.Fioranelli.pdf
13	https://dokumentarac.com/wp-content/uploads/2020/07/fioranelli.pdf
14	https://greatmountainpublishing.com/wp-content/uploads/2020/11/FIORANELLI-5G-Cause-of-COVID-19.pdf
15	https://www.orwell-news.ch/wp-content/uploads/2020/07/Fioranelli_5G-Technology-and-induction-of-coronavirus-in-skin-cells.pdf
16	https://jdfor2020.com/wp-content/uploads/2020/07/FIORANELLI.pdf
17	https://smombiegate.org/wp-content/uploads/2020/07/FIORANELLI.pdf
18	https://theageoftransitions.com/wp-content/uploads/2020/07/corona_virus_5g.pdf
19	https://www.synthesismeaning.me/s/5G_Technology_and_induction_of_coronavirus_in_skin_cells_by_M_Fioranelli.pdf
20	https://www.newbraveworld.org/wp-content/uploads/2020/07/5G_and_induction_of_coronavirus_in_skin_cells.pdf
21	https://z.zz.ht/0oIxa.pdf
22	http://wasserwandel.info/5G-Technology-and-induction-of-coronavirus-in-skin-cells.pdf
23	http://wake-up.acordem.com/downloads/fioranelli.pdf
24	http://domainofsatanists.uk/books/5G%20Technology%20and%20induction%20of%20coronavirus%20in%20skin%20cells.pdf
25	https://www.rumormillnews.com/pdfs/470581936-5G-Technology-and-Induction-of-Coronavirus-in-Skin-Cells1111.pdf
26	http://www.tanker-enemy.com/PDF/5G-Technology-and-induction-of-coronavirus-in-skin-cells-M.Fioranelli.pdf
27	http://alternativeprinciplesforhealth.info/wp-content/uploads/2020/08/fioranelli.pdf
28	https://search.bvsalud.org/global-literature-on-novel-coronavirus-2019-ncov/resource/en/covidwho-646363
29	https://www.stop5gticino.ch/wp-content/uploads/2020/08/5G-Technology-and-induction-of-coronavirus-in-skin-cells-Fioranelli.pdf

30	http://www.medicosporlaverdad-peru.com/wp-content/uploads/2020/11/5G_Technology_and_induction_of_coronavirus_in_skin_cells_by_M_Fioranelli.pdf
31	https://www.attitudeproductions.com/ANH/5G-Technology-and-Induction-of-Coronavirus-in-Skin-Cells.pdf (NB not marked “Copyright Biolife”, unlike most other versions available)
32	https://bürgerinitiative-5g-freies-köln.de/wp-content/uploads/2020/08/5G-Technology-and-Induction-of-Coronavirus-in-Skin-Cells1111.pdf (NB not marked “Copyright Biolife”, unlike most other versions available)
33	https://www.bearfootgrounding.com/sites/bearfootgrounding.com/files/FIORANELLI.pdf
34	https://mlpol.net/images/src/0280E86252F7E5DA1E5D3FE159B30534-1109451.pdf
35	https://www.emfhelpcenter.com/downloaddocs/5GCV.pdf
36	https://bewusst-klartext.org/wp-content/uploads/2020/07/Studie-FIORANELLI_2020_5G_Covid19.pdf
37	https://foundationfordisinfestation.com/wp-content/uploads/2020/09/FIORANELLI1.pdf
38	https://www.makrobiotikschweiz.ch/video_vortraege/pdf/5G-Technology-and-induction-of-coronavirus-in-skin-cells.pdf
39	https://bundesverband.rom-electronic.org/wp-content/uploads/2020/07/FIORANELLI.pdf
40	https://allamericanmag.com/wp-content/uploads/2020/07/5G-470042130-Fiora-Nell-i.pdf
41	https://dissidentsignposts.org/uploads/s/i/v/q/ivqvdwfeguyr/file/z96hYLSM.pdf
42	http://www.biblaridion.info/Anthology/5Geee.pdf
43	https://kundaliniandcelltowers.com/5G-Technology-and-induction-of-coronavirus-in-skin-cells-Journal-of-Biological-Regulators-and-Homeostatic-Agents-June-9-2020.pdf
44	https://ourfreesociety.com/5G/5G-technology-and-induction-of-coronavirus-in-skin-cells-Italy.pdf (NB not marked “Copyright Biolife”, unlike most other versions available)
45	https://fch.bet/.media/ad285aecc1927ae76382d7562553ac1bf245f98c74b1605be49676c20a55c7a6.pdf
46	http://rumbletalk-images-upload.s3.amazonaws.com/756410a89824d77318d63619dc68d210/1595595714-470042130-Fiora-Nell-i.pdf
47	http://www.mailaz.com/COVID-19/The_Document-5G.pdf
48	https://healthstronghold.com/wp-content/uploads/2020/08/FIORANELLI-COVID-5G.pdf
49	https://corona-transition.org/IMG/pdf/fioranelli_5g_technology_and_induction_of_coronavirus_in_skin_cells_470042130.pdf

50	https://www.thesilo.ca/wp-content/uploads/2020/07/5G-1.pdf
51	http://gamzuletova.org/wp-content/uploads/2020/07/FIORANELLI.pdf
52	http://gamzuletova.org/wp-content/uploads/2020/07/5G_InduceEndogenousCoronavirus-1.pdf
53	https://5gdangersemf.com/wp-content/uploads/2020/07/5G-And-Coronavirus.pdf
54	http://libertygalaxy.com/videos/pdf/FIORANELLI.pdf
55	https://www.transformation.dk/deling/5G_CAUSES_COVID.pdf
56	https://www.thesimplextruth.com/uploads/1/1/9/6/119697876/5g_technology_and_induction_of_coronavirus_in_skin_cells-fioranelli.pdf
57	https://www.millennium-products.com/wp-content/uploads/2020/07/5g-technology-coronavirus-skin-cells.pdf
**Full text sources no longer available at 26th June 2021**	
58	https://liberopensare.b-cdn.net/wp-content/uploads/2020/07/5G-Technology-and-induction-of-coronavirus.....pdf
59	https://www.beyondminds.org/archives/files/5g/5G%20Technology%20and%20induction%20of%20coronavirus%20in%20skin%20cells-FIORANELLI.pdf
60	http://reporteraustria.bplaced.net/wp-content/uploads/2020/09/FIORANELLI-1-2.pdf (no
**New full text sources available at 26th June 2021**	
1	https://bürgerinitiative-5g-freies-köln.de/wp-content/uploads/2020/08/5G-Technology-and-Induction-of-Coronavirus-in-Skin-Cells1111.pdf (NB not marked "Copyright Biolife", unlike most other versions available)
2	5G Technology and induction of coronavirus in skin cells – Choose Life (reformatted, unmarked, HTML version of original article)
3	https://gandhi-auftrag.de/FIORANELLI.pdf

**Unmarked abstracts remaining available at 26th June 2021**	
1	https://www.ka-electronics.com/images/pdf/5G_Technology_and_induction_of_coronavirus_in_skin_cells_PubMed.pdf
2	https://www.waynekirkwood.com/images/pdf/5G_Technology_and_induction_of_coronavirus_in_skin_cells_PubMed.pdf

3	http://factwrecker.com/pdf-pages/EMF/5G%20Technology%20and%20induction%20of%20coronavirus%20in%20skin%20cells%20-%20PubMed.pdf
